# Annexin A6-Induced Alterations in Cholesterol Transport and Caveolin Export from the Golgi Complex

**DOI:** 10.1111/j.1600-0854.2007.00640.x

**Published:** 2007-11

**Authors:** Laia Cubells, Sandra Vilà de Muga, Francesc Tebar, Peta Wood, Rachael Evans, Mercedes Ingelmo-Torres, Maria Calvo, Katharina Gaus, Albert Pol, Thomas Grewal, Carlos Enrich

**Affiliations:** 1 Departament de Biologia Cel·lular, Facultat de Medicina, Universitat de BarcelonaCasanova 143, 08036-Barcelona, Spain; 2 Centre for Immunology, St. Vincent’s Hospital, University of New South WalesSydney, NSW 2010, Australia; 3 Institut d’Investigacions Biomèdiques August Pi Sunyer (IDIBAPS), Facultat de Medicina, Universitat de BarcelonaBarcelona, Spain; 4 Unitat de Microscòpia Confocal, Serveis Cientificotècnics, Facultat de Medicina, Universitat de BarcelonaBarcelona, Spain; 5 Centre of Vascular Research, School of Medical Sciences, University of New South WalesSydney, NSW 2052, Australia

**Keywords:** annexin A6, caveolin, cholesterol, late endosomes, Golgi

## Abstract

Annexin A6 (AnxA6) belongs to a family of Ca^2+^-dependent membrane-binding proteins and is involved in the regulation of endocytic and exocytic pathways. We previously demonstrated that AnxA6 regulates receptor-mediated endocytosis and lysosomal targeting of low-density lipoproteins and translocates to cholesterol-enriched late endosomes (LE). As cholesterol modulates the membrane binding and the cellular location of AnxA6, but also affects the intracellular distribution of caveolin, we investigated the localization and trafficking of caveolin in AnxA6-expressing cells. Here, we show that cells expressing high levels of AnxA6 are characterized by an accumulation of caveolin-1 (cav-1) in the Golgi complex. This is associated with a sequestration of cholesterol in the LE and lower levels of cholesterol in the Golgi and the plasma membrane, both likely contributing to retention of caveolin in the Golgi apparatus and a reduced number of caveolae at the cell surface. Further strengthening these findings, knock down of AnxA6 and the ectopic expression of the Niemann–Pick C1 protein in AnxA6-overexpressing cells restore the cellular distribution of cav-1 and cholesterol, respectively. In summary, this study demonstrates that elevated expression levels of AnxA6 perturb the intracellular distribution of cholesterol, which indirectly inhibits the exit of caveolin from the Golgi complex.

Annexin A6 (AnxA6) is a member of a highly conserved family of Ca^2+^- and membrane-binding proteins that have been implicated in an array of physiological processes such as cell proliferation, differentiation and signal transduction (1,2). The different localization of each annexin in various cells and tissues analyzed and the interaction of individual annexins with different sets of membrane lipids and proteins, together with their association with cytoskeletal proteins, implicated a role for annexins in the regulation of membrane fusion, targeting and vesicle formation in endocytic and exocytic pathways (1,3). And indeed, in recent years, the involvement of annexins in the regulation of membrane traffic and membrane microdomain formation has emerged as one of their predominant functions. In many cells and tissues analyzed, AnxA6 was found both at the plasma membrane (PM) and in the early endosomes (EE) (4–6). These localization studies correlate with the stimulatory effect of ectopically expressed AnxA6 in receptor-mediated endocytosis (7–9) and favor a role of AnxA6 in the endocytic pathway.

Most cells and tissues express abundant amounts of AnxA6, and in rat liver, baby hamster kidney (BHK), normal rat kidney (NRK) cells and polarized WIF-B hepatocytes, immunocytochemical studies identified AnxA6 as a prominent component not only of EE but also of late endosomes (LE)/prelysosomes, Golgi compartments (6,10–12) and phagosomes (13). However, like all other annexins, AnxA6 is not expressed in some cell types and tissues analyzed (2,4). For example, epithelial cells of the small intestine and parathyroid gland and A431, a vulval squamous epithelial carcinoma cell line, completely lack AnxA6. Others, like colon epithelium, Chinese hamster ovary (CHO) cells and several human breast cancer cells have almost undetectable amounts of AnxA6 (2,8,14–16). These findings suggest cell-specific functions of AnxA6 that are associated with tightly controlled expression levels.

The regulation of the subcellular distribution of AnxA6 is poorly understood, but in close relation with its multiple locations and dynamic behavior, different and additional, yet unidentified signals seem to promote targeting of AnxA6 to certain cellular sites. Elevation of intracellular Ca^2+^ levels promotes the translocation of cytosolic AnxA6 to the PM and EE, where it is believed to bind to negatively charged phospholipids (1,17). However, in the late endosomal/prelysosomal compartment, recent findings from our laboratory suggest that cholesterol stimulates the membrane-binding affinity of AnxA6 (14). We identified that accumulation of low-density lipoproteins (LDL)-containing vesicles was accompanied by an increased amount of AnxA6 in LE (8). In addition, we showed that the expression of a dominant-negative mutant of AnxA6 inhibited the transport of LDL from the late endosomal/prelysosomal compartment to lysosomes (7). Finally, treatment with U18666A, a pharmacological agent that leads to an accumulation of cholesterol in LE, resulted in a significant and very specific translocation of both endogenous and ectopically expressed AnxA6 but not AnxA2 or caveolin in these vesicles *in vivo*(14). Although these mechanisms do not seem to operate in all cells and the healthy appearance of AnxA6 knockout (ko)-mice challenge the role of AnxA6 in this context (18,19), these studies indicate a role for AnxA6 in the lysosomal targeting and export of cholesterol from LE/prelysosomes.

Interestingly, studies on other proteins involved in the transport of cholesterol from LE/prelysosomes to other cellular compartments, such as Niemann–Pick C1 (NPC1), revealed that this process might be linked to the localization, trafficking and expression levels of caveolin-1 (cav-1) and caveolin-2 (cav-2) at the *trans*Golgi network (TGN) and at the PM (20–22). As AnxA6 in LE could be involved in the delivery of cholesterol to caveolin-containing compartments, we investigated its role in the subcellular localization and membrane trafficking of caveolin. In the present study, we demonstrate that increased expression of AnxA6 results in a sequestration and reduced export of cholesterol from LE, possibly by interacting and interfering with NPC1 function. This imbalance of intracellular cholesterol correlates with the accumulation of cav-1 in the Golgi complex and a reduced number of caveolae at the cell surface. The potential participation of AnxA6 in cholesterol transport from the late endocytic compartment, the mechanisms and its consequences for caveolin export from the Golgi complex are discussed.

## Results

### The AnxA6 is involved in cholesterol sequestration and export from LE

**Figure 5:**
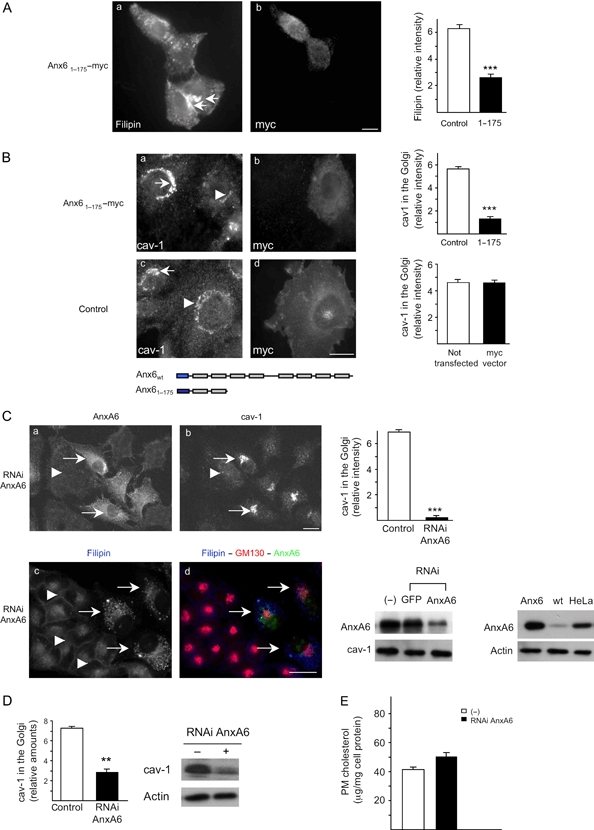
**Expression of an N-terminal deletion mutant or knock down of AnxA6 restores the cellular distribution of cholesterol and cav-1.**A) The CHOanx6 cells were transfected with Anx6_1–175_−myc (a and b), fixed, stained with filipin (5 μg/mL) (a) and immunolabeled with anti-myc (b) as indicated. Arrows point at vesicular, cholesterol-containing structures (a). Loss of strong perinuclear filipin staining was observed in 50% of transfected cells in five independent experiments and quantified. The mean ± SD of the relative filipin intensity in the perinuclear region is given. B) The CHOanx6 cells were transfected with Anx6_1–175_−myc (a and b) and empty vector (c and d). Twenty-four hours after transfection, cells were fixed, permeabilized and immunolabeled with anti-cav-1 (a and c) and anti-myc (b and d) as indicated. As shown in the schematic diagram, myc-tagged Anx6_1–175_ contains the N-terminal signal peptide (blue) and two out of eight AnxA6-membrane-binding repeats (gray). Arrows point at the increased amount of cav-1 in the Golgi of non-transfected and vector-transfected CHOanx6 cells (a and c). Arrowheads point at reduced and unchanged cav-1 staining at the Golgi in Anx6_1–175_-transfected (panel a) and control cells (panel c), respectively. Reduced cav-1 staining in the Golgi region upon Anx6_1–175_ expression was observed in 45–55% of transfected cells in five independent experiments. The fluorescence intensity of the cav-1 staining at the Golgi in Anx6_1–175_-transfected versus non-transfected (panel a) and myc-transfected cells (panel c) were quantified. Values represent the mean ± SD of 50 cells per cell line in each experiment (*n* = 5). ***, p < 0.001 for Student’s *t*-test. Bar is 10 μm. C) HeLa cells were transfected with RNAi targeting AnxA6 (RNAi–AnxA6) as described (15). Seventy-two hours after transfection, cells were fixed, permeabilized, immunolabeled with anti-annexin A6 (AnxA6), anti-cav-1 (cav-1) and anti-GM130 and stained with filipin as indicated. Arrowheads point at cells with downregulated AnxA6. Arrows show cells with unchanged AnxA6 expression levels and cav-1 staining (a and b) and vesicular filipin staining (c and d). In three independent experiments, reduced amounts of cav-1 in the Golgi region (a and b) and increased filipin staining at the PM (c and d) were observed in 60–70% of AnxA6-depleted cells. The fluorescence intensity of the cav-1 staining at the Golgi area in non-depleted/depleted cells was quantified. Values represent the mean ± SD of three independent experiments. ***, p < 0.001 for Student’s *t*-test. Expression levels of AnxA6 in CHOanx6 (Anx6), CHOwt (wt) and HeLa ± RNAi are given. Bar is 10 μm. D) HeLa cells were transfected with RNAi–AnxA6 as above. Seventy-two hours after transfection, Golgi–caveolin was immunoprecipitated as described in Figure. 4C. The relative amount of cav-1 in the Golgi was quantified and normalized to actin. Results represent the mean ± SD of three independent experiments with duplicate samples. **, p < 0.01 for Student’s *t*-test. E) The PM cholesterol (μg/mg cell protein) was determined as described above (Figure 2C) from HeLa cells transfected ± RNAi–AnxA6 (in triplicate) and normalized to cellular protein. The mean ± SD is given and is representative for two independent experiments.

To investigate the consequences of AnxA6 expression on the intracellular distribution of cholesterol, we first compared CHO wildtype (CHOwt) cells and a well-characterized CHO cell line overexpressing AnxA6 [(CHOanx6); see *Materials and Methods*for further details]. The CHOwt cells express very low amounts of AnxA6 (7,8,15,23) (see also Figures 5C and 8C and Figure S3C), whereas AnxA6 levels in CHOanx6 are rather similar to the AnxA6 expression observed in other commonly used cell lines such as HeLa (Figure 5C).

**Figure 1:**
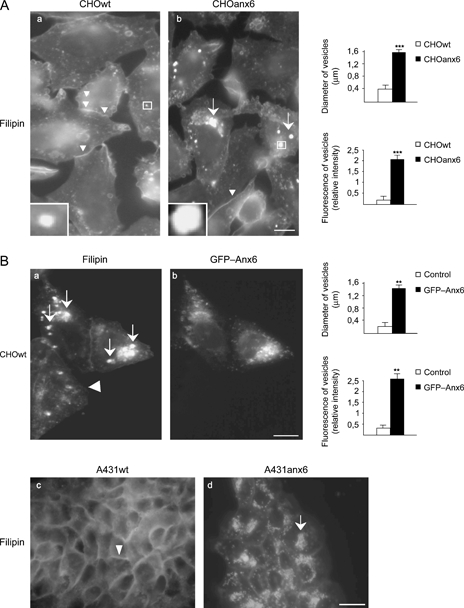

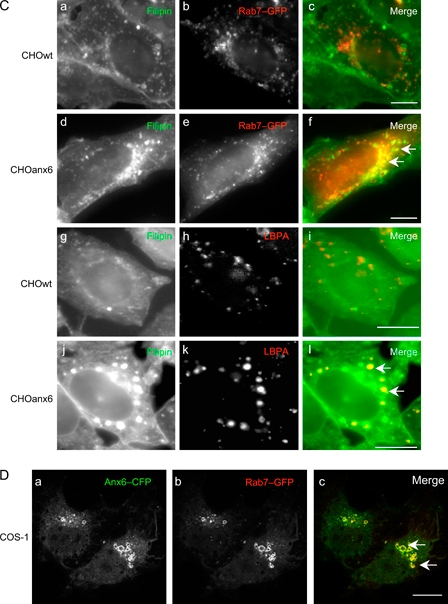
**Sequestration of cholesterol in the late endosomal compartment of AnxA6-expressing cells.**A) The CHOwt and CHOanx6 cells grown on coverslips were fixed and stained with filipin (5 μg/mL) as indicated. Arrows and arrowheads point at the accumulation of cholesterol in perinuclear compartments and at the PM, respectively. B) The CHOwt cells transiently transfected with GFP–Anx6 (a and b), AnxA6-deficient A431 cells (A431wt) and A431 cells stably expressing AnxA6 (A431anx6) were fixed and stained with filipin (c and d). The arrows point at the accumulation of cholesterol in perinuclear vesicles of GFP–Anx6 expressing CHO and A431anx6 cells, compared with the non-transfected control (arrowheads). For the quantification in A (a and b) and B (a and b), see text for further details. The mean values ± SD of the diameter and fluorescence intensity of perinuclear filipin-positive structures from three independent experiments (150 cells in total) per cell line is given; ** and ***, p < 0.01 and p < 0.001 for Student’s *t*-test, respectively. Inserts (in A) show representative filipin-positive structures. C) The CHOwt and CHOanx6 cells were transfected ± Rab7–GFP (a–c and d–f), fixed and immunolabeled with anti-LBPA (g–i and j–l) and stained with filipin (a, d, g and j) as indicated. The merged images (c, f, i and l) show a colocalization of cholesterol and LE in CHOanx6 cells (arrows in f and l). D) The COS-1 cells were cotransfected with Anx6–CFP (a) and Rab7–GFP (b), fixed and analyzed by confocal microscopy. Arrows in the merged image (panel c) point at colocalization of Anx6–CFP and Rab7–GFP. Bar is 50 μm in B (a and b) and 10 μm in all other panels. Figure 1 continued on next page.

The CHOwt and CHOanx6 cells were fixed, stained with filipin, and the pattern of cholesterol staining was compared by epifluorescence microscopy (Figure 1A). As expected, in CHOwt cells, cholesterol was clearly detectable at the PM, and in punctate, in part perinuclear, structures throughout the cytoplasm. In contrast, 90% of CHOanx6 cells showed a very different staining pattern; in particular, weak cholesterol staining at the PM and a much stronger accumulation of cholesterol in cytoplasmic, perinuclear vesicles (Figure 1A). Both the size and fluorescence intensity of filipin-stained vesicle cells were significantly increased in CHOanx6 compared with CHOwt cells (Figure 1A; p < 0.001). Quantification of only prominently enlarged cholesterol-containing structures (defined as cytoplasmic, filipin-positive vesicles with a Ø ≥ 0.5 μm and a relative intensity ≥0.4; see inserts and quantification in Figure 1A) confirmed an accumulation of cholesterol in 45.7 ± 14.6% of CHOanx6 cells with an average of 3.2 ± 0.7 filipin-positive perinuclear structures/cell. In contrast, only 14.3 ± 4.1% of control cells showed similar structures with an average of 1.3 ± 0.2 filipin-positive structures/cell (p < 0.001).

**Figure 8:**
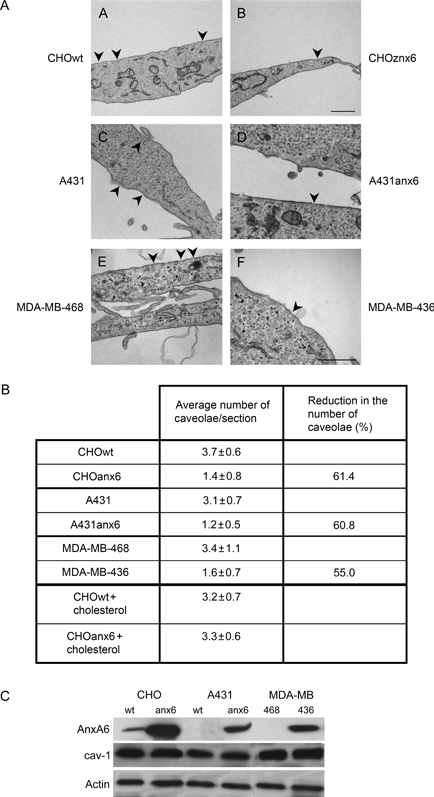
**High AnxA6 expression levels correlate with a reduced number of caveolae at the cell surface.**A) Monolayers of cells with low (CHOwt, A431wt and MDA-MB-468; panels a, c and e) and high (CHOanx6, A431anx6 and MDA-MB-436; panels b, d and f) AnxA6 expression levels (see Western blot analysis in C), or CHOwt and CHOanx6 cells preincubated with cholesterol were fixed, collected, embedded in Spurr, and ultrathin sections were analyzed by electron microscopy. Bar is 1 μm in panels a and b and 0.5 μm in c, d, e and f, respectively. B) Grids were systematically screened, and the number of caveolae (arrowheads in A) in each cell line was determined in random fields of sections from images acquired at the same magnification (25 000×). For each cell line and condition, over 50 cells were examined with an estimated total lineal surface of 500 μm. The average number of caveolae/section and the reduction in the number of caveolae (%) from three independent experiments are given. C) Expression levels of AnxA6, cav-1 and actin in the various cell lines analyzed above are given.

To validate the strikingly different distribution of cholesterol in the CHOanx6 cell line and to exclude an aberrant phenotype as a consequence of cell line selection, we then analyzed CHOwt cells transiently transfected with green fluorescent protein (GFP)-tagged AnxA6 (GFP–Anx6). In these set of experiments, and similar to the elevated levels of AnxA6 in the CHOanx6 cell line (11.1 ± 2.7-fold), expression levels of GFP–Anx6 were increased 9.4 ± 3.5-fold compared with endogenous AnxA6 in CHOwt cells (compare Figure S3B–C and Figures 5C and 8C). Clearly, transient overexpression of AnxA6 resulted in an accumulation of cholesterol in vesicular structures (Figure 1B; p < 0.01). The same results were obtained when the filipin staining in A431 (A431wt) cells, which completely lack endogenous AnxA6 (15,18), and A431 stably expressing AnxA6 [(A431anx6); Figure 1B, see also Figure 8C for AnxA6 expression levels] was compared. Thus, not the CHOanx6 cell line selection but elevation of AnxA6 levels *per se*correlates with an accumulation of cholesterol in vesicular structures. This is not because of the increased internalization rates as AnxA6 alone does not alter LDL endocytosis but requires the coexpression of the LDL receptor to increase LDL uptake and degradation (7,8). Similarly, high transferrin receptor coexpression revealed a stimulatory role of Rab5 in endocytosis (24,25). To identify if AnxA6-induced accumulations of cholesterol were in the late endocytic compartment, CHOwt and CHOanx6 cells were transfected with the LE marker GFP-tagged Rab7 (Rab7–GFP) and stained with filipin. This showed a substantial colocalization of Rab7–GFP and cholesterol in CHOanx6 cells (Figure 1C, panels a–f). In support of this, we also observed a significant colocalization of filipin with another marker for LE, lysobisphosphatidic acid (LBPA), only in CHOanx6 cells (Figure 1C, panels g–l) but not in controls. As shown previously in CHOanx6 and NRK cells (7, 14), significant amounts of AnxA6 colocalized with Rab7 in the late endosomal compartments of COS-1 cells transiently cotransfected with Anx6– cyan fluorescent protein (CFP) and Rab7–GFP (Figure 1D, panels a–c).

**Figure 2:**
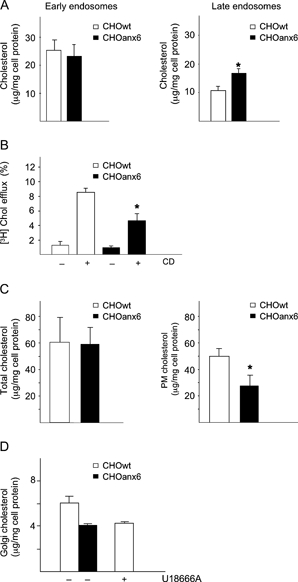
**Cholesterol imbalance in CHOanx6 cells.**A) The EE and LE from 4 to 6 × 10^7^ CHOwt and CHOanx6 cells were isolated as described (8,14) (see also Figure S1A), and cholesterol (μg/mg cell protein) in each endosomal fraction was determined in duplicate samples (see *Materials and Methods*). The mean ± SD is given and is representative for five independent experiments. *, p < 0.05 for Student’s *t*-test. B) The CHOwt and CHOanx6 cells were incubated with 2 μg/mL U18666A together with [^3^H]-Chol (2 × 10^6^ c.p.m/mL) for 24 h. Cells were washed with PBS and incubated ± 1% CD for 120 min. The ratio of released and cell-associated radioactivity was determined, normalized to total cell protein, and the amount of efflux is given in per cent. The background efflux in U18666A-treated CHOwt and CHOanx6 cells was equivalent to 5.0–7.0 × 10^5^ c.p.m/mg cell protein, respectively. Mean values ± SD of three independent experiments with triplicate samples are given. *, p < 0.05 for Student’s *t*-test. C) Total cellular cholesterol and PM cholesterol (μg/mg cell protein) were determined from whole cell lysates and PM fractions (15) (see also Figure S1B) of CHOwt and CHOanx6 cells (in triplicate) and normalized to cellular protein. The mean ± SD is given and is representative for three and five independent experiments, respectively. *, p < 0.05 for Student’s *t*-test. D) The CHOwt cells were incubated ± U18666A (2 μg/mL) for 24 h as indicated, and cholesterol levels in 500 μL of Golgi-enriched membrane fractions were determined and compared with CHOanx6 cells. Mean values ± SD of two independent experiments with duplicate samples are given.

To confirm that expression of AnxA6 increases the content of cholesterol in LE by biochemical means, cell lysates from CHOwt and CHOanx6 cells were subjected to cell fractionation, and EE and LE were isolated on sucrose gradients (8) and analyzed for their cholesterol (Figure 2A) and phospholipid contents. The purity of isolated fractions was routinely assessed by immunoblotting with established markers for EE (Rab5 and EEA1), LE (Rab7 and Lamp1), PM [Na^+^K^+^ adenosine triphosphatase (ATPase)] and β-hexosaminidase activity assays (lysosomes) as described (8,14) (Figure S1A). Similar to previous studies (14), high-performance liquid chromatography analysis and fluorometric determinations showed comparable levels of cholesterol (Figure 2A) and phospholipids (phosphatidylglycerol, phosphatidylethanolamine, phosphatidylinositol, phosphatidylserine (PS) and phosphatidylcholine; unpublished data) in EE fractions of both cell lines. In contrast, and in agreement with the increased filipin staining (Figure 1A–C), LE from CHOanx6 cells contained more cholesterol (55 ± 12%; p < 0.01), whereas the molar ratio of phospholipids remained unchanged. The differences in late endosomal cholesterol levels measured biochemically in CHOanx6 cells versus CHOwt cells (Figure 2A) appear to be smaller than the differences in filipin staining (Figure 1A–C) between these cells. However, these results are consistent with the reports showing that moderate increases (30–40%) in late endosomal cholesterol result in a dramatic intensification of filipin staining (26–29).

Late endosomal/prelysosomal cholesterol is exported to various locations in the cell including the cell surface. In normal cells at steady state, very little cholesterol is detected in LE and lysosomes as cholesterol derived from the hydrolysis of internalized LDL is rapidly transported out of LE (30–32). Thus, cholesterol export from LE cannot easily be quantified as incubation of cells with radioactive cholesterol {[^3^H]-Cholesterol ([^3^H]-Chol)} preferentially labels the PM and to a lesser extent other intracellular compartments such as recycling endosomes and the Golgi. However, in the presence of U18666A, [^3^H]-Chol almost exclusively loads LE, and followed by incubation with cholesterol acceptors like methyl-β-cyclodextrin (CD), [^3^H]-Chol from LE is available for efflux to the PM and can be quantified (33,34). Although the CD-induced export of cholesterol is significantly delayed under these conditions and approximately fivefold to sevenfold less efficient as in control CHO cells (33) (see also Figure S2A), it allows to determine if cholesterol export from LE is affected upon AnxA6 overexpression. The CHOwt and CHOanx6 cells were incubated with U18666A in the presence of [^3^H]-Chol, followed by 1% CD for 120 min, and then the radioactivity released into the media was measured (Figure 2B). Compared with controls, CHOanx6 cells showed a 53.9 ± 11.5% reduction (p < 0.05) in cholesterol efflux upon incubation with CD. Similar results were obtained when comparing other cell lines (A431 and BT20) ± AnxA6 (Figure S2B). Consistent with these findings, and despite the total amount of free cholesterol being comparable in both cell lines (Figure 2C), the cholesterol in isolated PM fractions was significantly reduced by 42 ± 12% (p < 0.05) in CHOanx6 cells (Figure 2C). Cholesterol efflux induced with 1% CD or physiological cholesterol acceptors [high-density lipoproteins (HDL), HDL_3_] in the absence of U18666A, which will then predominantly remove [^3^H]-Chol derived from the PM, was reduced by 10–20% in CHOanx6 cells (Figure S2A). Although we cannot completely rule out some contribution of efflux from intracellular compartments in these experiments, it is supportive of cholesterol at the PM being reduced in CHOanx6 cells.

To address if cholesterol synthesis in AnxA6-expressing cells could contribute to the reduced cholesterol levels at the PM, CHOwt and CHOanx6 cells were labeled with [1-^14^C]-acetic acid. Newly synthesized radioactive cholesterol was separated from its major precursors and analyzed by thin layer chromatography (TLC). The TLC plates were exposed to X-ray film, quantified using ImageJ [National Institutes of Health (NIH)] and normalized for total cellular protein as described (35). Interestingly, in two separate experiments with triplicate samples, relative cholesterol synthesis was slightly reduced by 36 ± 18% (p < 0.05) in CHOanx6 cells (Figure S1B). Similar findings have been made for the caveolin mutant cavDGV (21) and could be (i) because of a reduced expression/activity of cholesterol synthetic enzymes in CHOanx6 cells or (ii) linked to inhibitory effects of AnxA6 and cavDGV on the Ras/PI3K/Akt signaling pathway in cholesterol synthesis (15,23,35,36). However, although newly synthesized cholesterol from the endoplasmic reticulam (ER) rapidly reaches the cell surface (37), it is unlikely that this pathway plays a major role in the reduction of cholesterol levels at the PM (and the Golgi; Figure 2D) of CHOanx6 cells as the majority of cellular cholesterol is derived from receptor-mediated uptake of lipoproteins, which is accompanied by a downregulation of cholesterol synthesis (31,32,37). In further support of this observation, the total amount of cellular cholesterol remains unchanged in CHOanx6 cells (Figure 2C).

Finally, to analyze if AnxA6-dependent reduction of cholesterol export from LE could be linked to cholesterol-dependent secretory pathways from the Golgi to the PM, we compared cholesterol levels in Golgi-enriched membrane fractions of CHOwt cells ± U18666A with untreated CHOanx6 cells. The purity of isolated Golgi-enriched membrane fractions was confirmed through the enrichment of *cis*- and *trans*-Golgi markers [(GM130, Golgi microtubule-associated protein of 210kDa (GMAP210) and TGN38) and the lack of significant amounts of EE, LE (Rab5 and Rab7), PM (Na^+^K^+^ ATPase) or endoplasmic reticulum (KDEL) markers (Figure S1C). As shown in Figure 2D, isolated CHOwt cells Golgi membranes contain 6.0 ± 0.4 μg cholesterol/mg cell protein, which is comparable to previously reported values (38,39). In contrast, CHOanx6 cells showed reduced (38.0 ± 5.7%) cholesterol levels in their Golgi fractions. Importantly, upon treatment of CHOwt cells with U18666A, reduced cholesterol levels in Golgi fractions were observed, which were comparable to the values obtained from untreated CHOanx6 cells. As newly synthesized cholesterol mostly bypasses the Golgi complex on its way to the PM, and the redistribution of internalized cholesterol from LE is the major source of cholesterol in the Golgi (37), these findings indicate that the blockage of cholesterol export from LE in U18666A-treated CHO cells mimics the effects observed in AnxA6-expressing cells, both ultimately leading to an impaired supply of cholesterol to the Golgi.

In summary, we conclude that AnxA6 is involved in cholesterol export from LE, and possibly in cholesterol synthesis, thereby contributing to an imbalance and altered distribution of cholesterol in other compartments like the Golgi apparatus and the cell surface.

### Expression of AnxA6 leads to the accumulation of caveolin in the Golgi

**Figure 3:**
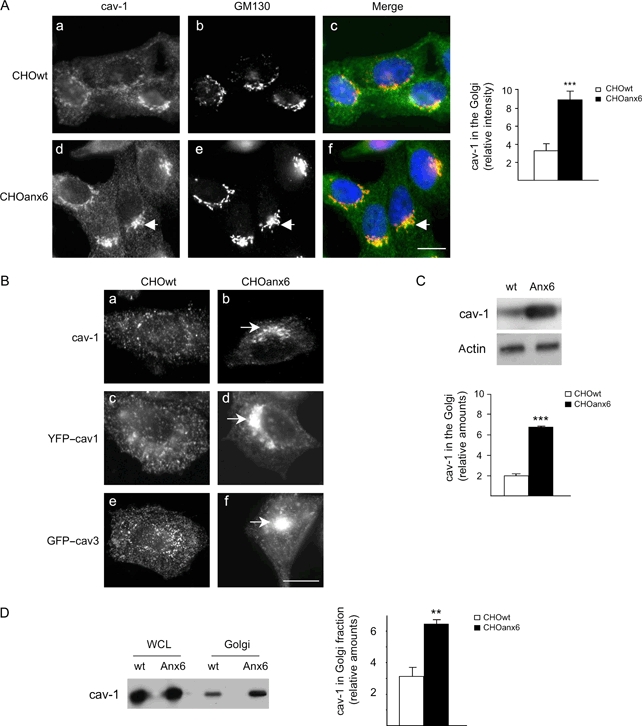
**Caveolin accumulates in the *cis*-Golgi compartment of AnxA6-expressing cells.**A) The CHOwt (a–c) and AnxA6-expressing CHO cells (CHOanx6, d–f) grown on coverslips were fixed, permeabilized and stained with polyclonal anti-caveolin (green), anti-GM130 (red) and 4,6-diamidino-2-phenylindole dihydrochloride (nuclei, blue) as indicated. The arrow points at caveolin (cav-1) accumulation and colocalization with GM130 in the Golgi of CHOanx6 cells. Images, focused at the plane of the Golgi, are representative for 90% of cells analyzed in each experiment (*n* = 5). Bar is 10 μm. The fluorescence intensity of the cav-1 staining at the Golgi area in CHOwt and CHOanx6 cells was quantified. Values represent the mean ± SD of five independent experiments with 50 cells per cell line in each experiment. ***, p < 0.001 for Student’s *t*-test. B) (a and b) The CHOwt and CHOanx6 cells were fixed, permeabilized and immunolabeled with mouse anti-cav-1 that specifically recognizes the Golgi-associated pool of cav-1. (c–f) The CHOwt and CHOanx6 cells were transfected with YFP–cav1 and GFP–cav3 as indicated. Twenty-four hours after transfection, cells were fixed, and the localization of YFP–cav1 and GFP–cav3 was analyzed by epifluorescence microscopy. Arrows point at the perinuclear accumulation of endogenous cav-1 (b) and transfected YFP–cav1 (d) and GFP–cav3 (f) in CHOanx6 cells, respectively. Images are representative for 70–80% of cells analyzed in five independent experiments. C) Golgi-associated caveolin was immunoprecipitated, analyzed by Western blotting as described (40) and normalized to actin. The relative amount of cav-1 in the Golgi in both cell types is given and represents the mean ± SD of five independent experiments with duplicate samples. ***, p < 0.001 for Student’s *t*-test. D) Protein levels of caveolin in whole cell lysates (WCL) and Golgi-enriched fractions (Golgi; see also Figure S1C) of CHOwt and CHOanx6 cells were determined by Western blot analysis and quantified. Mean values ± SD of three independent experiments with duplicate samples are given. **, p < 0.01 for Student’s *t*-test.

We have recently shown that cholesterol regulates the transport of caveolin through the Golgi complex (40). As the export of cholesterol from LE could be linked to caveolin trafficking and given the accumulation of late endosomal cholesterol in CHOanx6 cells, we first compared the localization of cav-1 in CHOwt cells and CHOanx6 cells (Figure S3A). Both cell types express very similar amounts of cav-1 and other proteins commonly found in caveolae such as Scavenger receptor BI (SR-BI) and H-Ras (15) (Figure S3C). Similar to other cell types, cav-1 is localized at the PM and in the intracellular compartments in CHOwt cells (images were taken with the focus at the plane of the Golgi). However, in more than 90% of AnxA6-expressing cells, a significant accumulation of caveolin in the perinuclear region was observed. Quantification of cav-1 fluorescence confirmed a 4.5 ± 0.4-fold (p < 0.001) increase of caveolin staining in the perinuclear (Golgi) region of CHOanx6 cells compared with controls (Figure S3A). To validate these findings, the localization of cav-1 in CHOwt cells transiently transfected with GFP–Anx6 was analyzed (Figure S3B). Similarly, cav-1 accumulated 4.5 ± 0.5-fold (p < 0.01) in the perinuclear region of CHOwt cells upon GFP–Anx6 overexpression. Double immunolabeling with polyclonal anti-caveolin and anti-GM130, a marker protein for the *cis*-Golgi compartment, revealed a remarkable colocalization in CHOanx6 cells (Figure 3A, panel f). Quantification of cav-1 fluorescence showed a 2.7 ± 0.4-fold (p < 0.001) increase of caveolin in the GM130-containing compartment of CHOanx6 cells (Figure 3A). Comparable data were obtained using a monoclonal antibody specifically recognizing Golgi-associated cav-1 (40) (Figure 3B, panels a and b).

Several caveolin antibodies preferentially recognize the Golgi but not the PM pool of caveolin (40). To rule out that expression of AnxA6 interfered with the antibody detection of caveolin at the PM, CHOwt and CHOanx6 cells were transiently transfected with yellow fluorescent protein (YFP)-tagged cav-1 (YFP–cav1) or GFP-tagged caveolin-3 (GFP–cav3) and visualized by epifluorescence microscopy (Figure 3B, panels c–f). As shown for endogenous cav-1, YFP–cav1 and GFP–cav3 were found in intracellular compartments and in the PM in CHOwt cells. In contrast, the majority of transfected CHOanx6 cells (70–80%) were characterized by a strong accumulation of both YFP–cav1 and GFP–cav3 in the perinuclear region, further indicating a retention or slow export of caveolin out of the Golgi complex.

**Figure 4:**
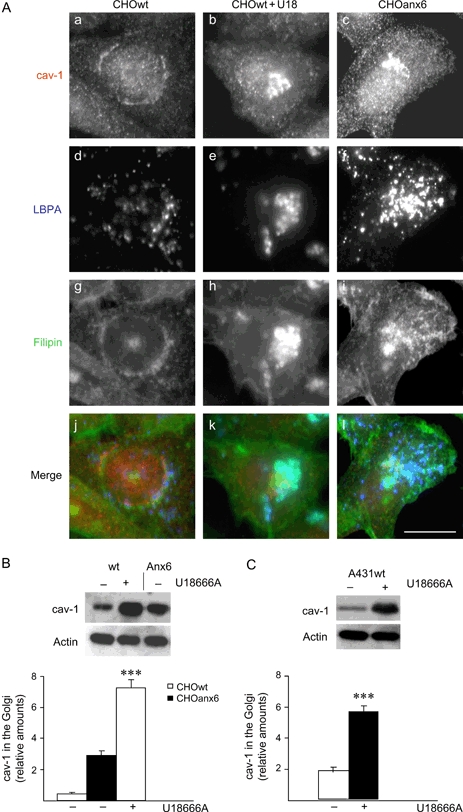
**Sequestration of cholesterol in LE alters the cellular distribution of caveolin.**A) The CHOwt and CHOanx6 cells were treated ± U18666A (2 μg/mL) for 24 h, fixed and stained with anti-caveolin, anti-LBPA and filipin (5 μg/mL) as indicated. A redistribution and increased cav-1 staining in the Golgi of U18666A-treated CHOwt can be observed (panels b and n). The merged images are shown (panels j–l). Bar is 10 μm. B and C) The CHOwt, CHOanx6 (B) and A431 (C) cells were treated ± U18666A as above, and Golgi-associated caveolin was immunoprecipitated and analyzed by Western blotting as described (40). Actin in the cell lysates is shown. The amount of immunoprecipitated cav-1 from the Golgi was quantified and normalized to actin. The relative amount of cav-1 in the Golgi is given and is representative for three independent experiments. ***, p < 0.001 for Student’s *t*-test.

By means of a biochemical approach developed recently in our laboratory (40), the amount of cav-1 in the Golgi was compared between both cell lines in immunoprecipitation experiments (Figure 3C). These experiments confirmed the increase of Golgi-associated cav-1 in CHOanx6 cells (3.4 ± 0.2-fold; p < 0.0001; see also Figure 4B and Figure S4B). The Cav-1 immunoprecipitates did not contain detectable amounts of AnxA6. Together with the lack of colocalization of AnxA6 with cav-1 in Golgi compartments (data not shown), this indicates that the increased association of cav-1 with the Golgi in CHOanx6 cells is not because of a direct interaction of AnxA6 with cav-1. In support of the results described above, when Golgi fractions from CHOwt and CHOanx6 cells were purified and analyzed for the amount of cav-1, we identified a 2.8 ± 0.5-fold (p < 0.001) increase of cav-1 in Golgi fractions from CHOanx6 cells (Figure 4D).

The CHOwt cells express small amounts of AnxA6 making it difficult to detect in LE (14). To identify only residual amounts of endogenous AnxA6 in LE of U18666A-treated CHOwt cells, purified LE fractions had to be concentrated 10-fold (14). Similar to RNA interference (RNAi) knockdown approaches (see also Figure 5C), we reasoned that this very low concentration of AnxA6 in LE makes CHOwt cells an appropriate model system to analyze if sequestration of cholesterol in LE *per se*can already lead to an accumulation of cav-1 in the Golgi. Therefore, we first compared the localization of cav-1 in CHOwt cells ± U18666A (Figure 4A). As expected, CHOwt cells incubated with U18666A are characterized by increased amounts of cholesterol in LE (Figure 4A, compare panels g and h). In confirmation of previous results (14), treatment with U18666A did not result in the recruitment of cav-1 to LE, but a redistribution and increased cav-1 staining in the Golgi in CHOwt cells were observed (compare panels a and b). This correlates with a 4.1 ± 2.3-fold enlargement of LE in CHOwt cells upon drug treatment (compare panels d and e), which can already be observed in untreated CHOanx6 cells (panel f). It should be noted that CHOanx6 contained 2.5 ± 0.3-fold more LBPA-positive vesicles compared with CHOwt cells (compare panels d and f), indicating a potential enlargement of the late endosomal compartment. In support of these findings, immunoprecipitation of Golgi-associated cav-1 in CHOwt cells treated with U18666A was increased 17.7 ± 0.7-fold (p < 0.001) compared with untreated CHOwt controls (Figure 4B). To ensure that sequestration of cholesterol in LE in an AnxA6-independent manner can result in an accumulation of cav-1 in the Golgi, we analyzed an AnxA6 ko-model using A431 (A431wt) cells (15,18). The A431wt were treated with U18666A, and the Golgi-associated cav-1 was immunoprecipitated and analyzed by Western blotting as described above (Figure 4C). Like CHOwt cells (Figure 4B), A431 cells showed a 2.6 ± 0.4-fold (p < 0.0001) increase of cav-1 in the Golgi upon treatment with U18666A. These findings suggest that the accumulation of late endosomal cholesterol, possibly accompanied by LE vesicle enlargement as observed here and proposed by others (41), leads to the concomitant retention of caveolin in the Golgi. In summary, the results described above suggest that AnxA6 is a component of the molecular machinery involved in cholesterol homeostasis that indirectly modulates the export of cav-1 from the Golgi.

### Downregulation of AnxA6 restores the intracellular distribution of cholesterol and caveolin

We then transiently transfected CHOanx6 cells with a myc-tagged N-terminal deletion AnxA6 mutant (previously described as dominant-negative DN-anx6_1–175_) (Figure 5A), which interferes with the function of endogenous AnxA6 in the endocytosis and delivery of LDL to lysosomes (7, 9). This mutant (Anx6_1–175_) also seems to interfere with the function of ectopically expressed AnxA6 as observed by the loss of the characteristic distribution, size and number of enlarged, perinuclear filipin-positive structures (Figure 5A, arrows in panel a; loss of vesicular filipin-intensity in Anx6_1–175_-transfected CHOanx6 cells: 2.5 ± 0.5-fold; p < 0.0001). Furthermore, whereas cav-1 accumulates in the Golgi of CHOanx6 cells (Figure 5B, arrows in panels a and c; see also Figures 3 and 4), expression of the Anx6_1–175_ mutant (Figure 5B, arrowhead in panel a) strongly reduced the amounts of cav-1 at the Golgi in 45–55% of transfected cells (loss of caveolin staining in the Golgi in Anx6_1–175_-transfected CHOanx6 cells: 4.3 ± 0.3-fold; p < 0.0001). In contrast, CHOanx6 transfected with myc-tagged vector control (Figure 5B, panels c and d) still show increased cav-1 staining at the Golgi (arrowhead in panel c).

Then we analyzed the localization of cav-1 in AnxA6-depleted HeLa cells, which normally express AnxA6 levels, as shown for CHOanx6 cells that are significantly higher (8.2 ± 1.8-fold) compared with CHOwt cells (Figure 5C). The RNAi-mediated downregulation of AnxA6 by 70–80% did not affect cav-1 expression levels, but Golgi-cav-1 staining was strongly reduced (24.5 ± 0.21-fold; p < 0.001) in the majority (60–70%) of AnxA6-depleted cells (arrowheads in Figure 5C) as compared with the untransfected (arrows) or RNAi–GFP-transfected controls (not shown). Next, we determined the amounts of immunoprecipitated and Golgi-associated cav-1 after RNAi-mediated depletion of AnxA6 (Figure 5D). In agreement with the data obtained from the immunofluorescence microscopy, Golgi-associated cav-1 in AnxA6-depleted HeLa cells was reduced 2.6 ± 0.3-fold (p < 0.001). We then analyzed the distribution of cholesterol in AnxA6-depleted HeLa cells (Figure 5C). In AnxA6-expressing controls, a strong vesicular filipin staining was observed (arrows). In contrast, AnxA6 depletion resulted in a redistribution of cellular cholesterol with filipin predominantly staining the PM and the perinuclear GM130-positive region (arrowheads). This correlates with increased cholesterol levels (25 ± 3.5%) in PM-enriched membrane fractions of AnxA6-depleted cells (Figure 5E). Interestingly, depletion of Rab9, which upon overexpression can compensate for the accumulation of cholesterol in NPC-mutant cells (42–44), does not change the filipin pattern of HeLa cells (45). This indicates that more than a single pathway regulates cholesterol export from LE and proteins like AnxA6 and Rab9 drive efflux through different pathways. In summary, loss or downregulation of AnxA6 correlates with a redistribution of cholesterol, thereby modulating the intracellular transport of cav-1 from the Golgi to other cellular compartments.

**Figure 6:**
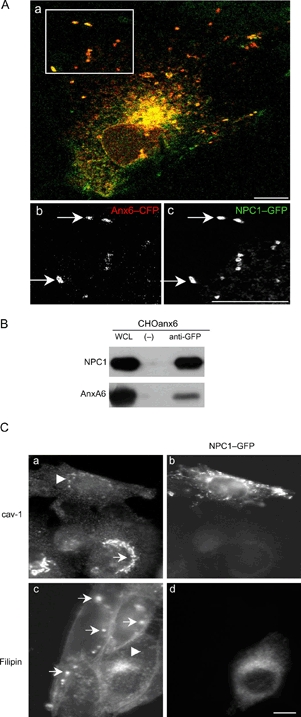
**The NPC1 restores the cellular distribution of cholesterol and cav-1 in AnxA6-overexpressing cells.**A) The COS-1 cells were cotransfected with Anx6–CFP (b) and NPC1–GFP (c), fixed, and analyzed by confocal microscopy. The merged image, which is representative for 90% of cells analyzed, is shown in panel a. Arrows in the enlarged sections (panels b and c) point at colocalization of AnxA6ndash;CFP and NPC1–GFP. Bar is 10 μm. B) The CHOanx6 cells were transfected with NPC1–GFP. Twenty four hours after transfection, cell lysates (WCL) were immunoprecipitated with or without (−) a polyclonal antibody against GFP (anti-GFP) as described (15) and analyzed by Western blotting for the presence of NPC1–GFP and AnxA6. C) The CHOanx6 cells were transfected with NPC1–GFP (b and d), fixed, permeabilized and immunolabeled with anti-caveolin (a) and stained with filipin (c). Cav-1 and cholesterol accumulation (arrows) in the Golgi and LE (panels a and c) is lost upon overexpression of NPC1 (arrowheads in panels a and c). Bar is 10 μm.

U18666A-treated CHOwt cells and AnxA6-expressing cells show a similar phenotype that partially resembles alterations in cholesterol transport, observed in cells carrying a mutation in the NPC1 protein. Given these similarities and the predominant localization of NPC1 in LE, we investigated if AnxA6 is linked to the function/localization of NPC1. Immunofluorescence microscopy revealed the colocalization of Anx6–CFP and GFP-tagged, wildtype NPC1 (NPC1–GFP) in transiently transfected COS-1 cells (Figure 6A). In addition, AnxA6 co-immunoprecipitated with NPC1–GFP in lysates from transfected CHOanx6 cells (Figure 6B). Most importantly, ectopic expression of NPC1–GFP in CHOanx6 cells resulted in a redistribution of cav-1 (Figure 6C, panel a) and cholesterol (panel c), indicating that overexpression of NPC1 overcomes the inhibitory effect of AnxA6 on cholesterol export in LE, thereby also releasing the blockage of cav-1 export from the Golgi (Figure 6C).

### The AnxA6 modulates the export of caveolin from the Golgi complex

**Figure 7:**
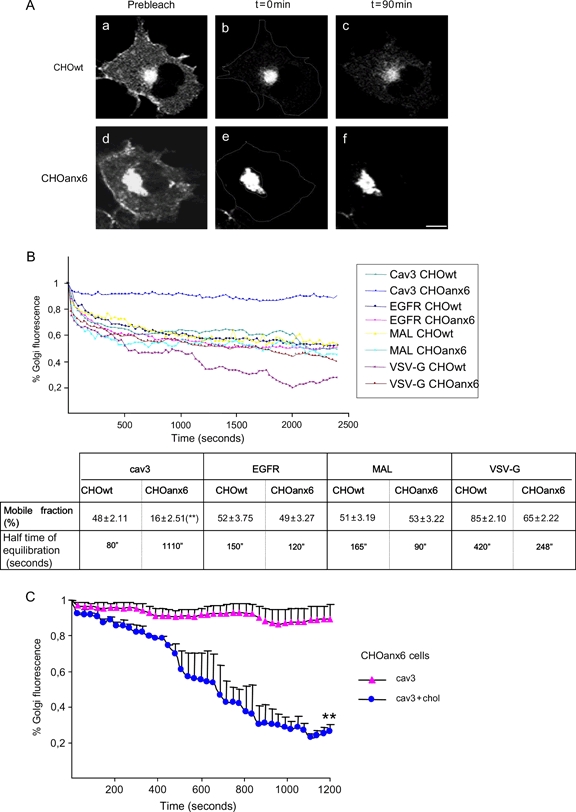
**Annexin A6 modulates the export of caveolin from the Golgi.**A) The CHOwt and CHOanx6 cells were transfected with GFP–cav3 and imaged before and after photobleaching of the entire cell except for the Golgi region. The loss of fluorescence signal (iFRAP) from the Golgi was monitored by time-lapse microscopy. To avoid fluorescence recovery from the novo synthesis, cells were treated with Cyhx. Representative Golgi images from CHOwt (a–c) and CHOanx6 (d–f) cells show GFP–cav3 localization before (a and d) and after bleaching at t = 0 min (b and e) and t = 90 min (c and f), respectively. A representative image from four independent experiments is shown. Bar is 10 μm. B) Quantification of Golgi iFRAP kinetics of GFP–cav3, EGFR–GFP, GFP–MAL and VSV-G–GFP in transfected CHOwt and CHOanx6 cells. Images were taken every 30 seconds (see *Materials and Methods*, t = 0–2500 seconds). Kinetic parameters (mobile fraction in % ± SD and half time of equilibration in seconds) for each protein in both cell lines from four independent experiments are given. **, p < 0.01 for Student’s *t*-test. C) Quantification of Golgi iFRAP kinetics of CHOanx6 cells transfected with GFP–cav3 as described in A and incubated ± cholesterol for 20 min as indicated. Images were taken every 30 seconds. The amount of the mobile fraction ± SD (%) in the Golgi is shown.

Given the requirement of cholesterol for caveolin export from the Golgi (40) and the reduced Golgi–cholesterol in CHOanx6 cells (Figure 2D), we determined the export of caveolin from the Golgi complex of CHOwt and CHOanx6 cells in inverse fluorescence recovery after photobleaching (iFRAP) experiments (40). In order to measure and visualize the kinetics of caveolin export, and to avoid any interference of GFP-tagged cav-1 with endogenous cav-1, we monitored the localization of GFP-tagged caveolin-3 (cav-3) (GFP–cav3). We and others have shown that the localization and trafficking of these two isoforms are comparable in commonly used cell lines that do not express cav-3 (36,40). Therefore, CHOwt and CHOanx6 cells were transfected with GFP–cav3, and the entire cytoplasm except the Golgi region of transfected cells was photobleached. Then GFP–cav3 trafficking from the Golgi was monitored by confocal microscopy. Results from individual cells were compared, and a representative experiment is shown (Figure 7A, for quantification, see Figure 7B). As hypothesized, 90 min after photobleaching, 48 ± 2.11% of GFP–cav3 fluorescence disappeared from the Golgi in CHOwt cells (mobile fraction, for quantification details, see *Materials and Methods*), but much slower kinetics for GFP–cav3 export (mobile fraction, 16 ± 2.51%) from the Golgi were observed in AnxA6-expressing cells (p < 0.01). Moreover, this appeared to be specific for caveolin as the kinetics of GFP–MAL, a raft-associated integral membrane protein at the PM, and GFP-tagged epidermal growth factor receptor (EGFR–GFP), which is found at the PM in both lipid rafts and clathrin-coated pits, were almost identical in CHOanx6 and controls (Figure 7B). Similar to published data (40,46), the kinetics and cholesterol requirements for the export of vesicular stomatitis virus-G protein [(VSV-G)–GFP], a marker protein for the exocytic pathway, are in striking contrast to cav-1 (see also Figure S5). Elevation of cholesterol leads to a retention of VSV-G in the Golgi, which was observed in both CHOwt and CHOanx6 cells. Consistent with our hypothesis of an impaired supply of cholesterol to the Golgi in CHOanx6, one could expect an increased mobility of Golgi-associated VSV-G. Indeed, in the set of experiments performed (Figure 7B), we observed a 20 ± 2% increased mobility of Golgi-associated VSV-G in CHOanx6 cells compared with controls.

We have recently demonstrated that exogenous cholesterol stimulates the exit of cav-1 from the Golgi (40). To determine if the accumulation and slow transit of caveolin through the Golgi in CHOanx6 cells could be overcome by cholesterol loading, iFRAP experiments ± exogenous cholesterol in CHOanx6 cells transiently transfected with GFP–cav3, which behaves like cav-1 in CHOanx6 (Figures 3B and 7A,B), were performed, and the kinetics of GFP–cav3 export were determined (Figure 7C). Clearly, addition of exogenous cholesterol completely re-established the onward trafficking of GFP–cav3 out of the Golgi complex in CHOanx6 cells *in vivo*. Then, the localization of endogenous cav-1 in controls and CHOanx6 cells ± cholesterol was compared (Figure S4A). Similarly, significantly less cav-1 was associated with the Golgi complex upon addition of cholesterol, but PM staining of cav-1 was evidently detectable in CHOanx6 cells (arrow in Figure S4A, panel d).

To corroborate these findings, Golgi-associated cav-1 was quantified in immunoprecipitation experiments (Figure S4B). The CHOwt and CHOanx6 cells were again incubated ± cholesterol for 90 min. Then the Golgi-associated cav-1 was immunoprecipitated and quantified as described (40) (see also Figures 3C, 4B,C and 5D). Control samples confirmed increased amounts of cav-1 associated with the Golgi complex in CHOanx6 cells (Figure S4B, compare lanes 1 and 5 and 2 and 6, respectively; p < 0.001). Addition of exogenous cholesterol reduced the amount of cav-1 in the Golgi by 37 and 84% in CHOwt cells as well as 63 and 68% in CHOanx6 cells ± cycloheximide (Cyhx), respectively (Figure S4B; p < 0.001).

Finally, to verify and identify a possible correlation between the level of AnxA6 expression, the accumulation of cav-1 in the Golgi and the number of caveolae at the cell surface, several cell types expressing high and low levels of AnxA6 were analyzed and compared by electron microscopy (Figure 8A). The CHOanx6 cells had morphologically normal but reduced numbers (61.4%) of caveolae compared with control cells (Figure 8B). Less caveolae (60.8%) were also found in A431 stably expressing AnxA6 (A431anx6) compared with AnxA6-deficient A431wt. Likewise, the comparison of estrogen-receptor (ER) negative breast cancer cell lines with high (MDA-MB-436) and low (MDA-MB-468) AnxA6 levels showed a 55.0% reduction in the number of caveolae in the ER-negative cells with high AnxA6 expression levels. In addition, exogenous cholesterol increased caveolae formation in CHOanx6 cells and resulted in comparable numbers of caveolae in both cell types (3.19 ± 0.68 and 3.32 ± 0.59 caveolae/section, respectively). These findings correlated with a slight decrease of HDL_3_-induced cholesterol efflux, which is believed to be mediated through SR-BI in caveolae [(47) and references therein], in CHOanx6 cells (10–20%) compared with controls. Similar results were obtained when cholesterol efflux was determined using 1% CD (Figure S2A). Thus, we conclude that the overall ability of CHOanx6 and other cell types with high AnxA6 expression levels to form caveolae is not affected. In spite of this, AnxA6-induced perturbations in cholesterol metabolism, including late endosomal cholesterol export indirectly reducing the exit of cav-1 from Golgi compartments, could contribute to a reduced number of caveolae at the cell surface. These results are also consistent with the reduced number of caveolae observed in BHK cells treated with U18666A (A. Pol and R. G. Parton, personal communication).

## Discussion

In this study, we have shown that high expression levels of AnxA6 can induce a cholesterol imbalance, thereby indirectly perturbating cav-1 export from the Golgi.

To our knowledge, only studies with (i) a dominant-negative mutant of cav3, cavDGV, and (ii) mutants of the NPC1 protein have shown a phenotype similar to AnxA6-expressing cells and revealed a link between intracellular cholesterol pools and caveolin localization.

Localization studies of N-terminal caveolin deletion mutants, including cavDGV, identified the C-terminal cytoplasmic domain being responsible for the targeting of caveolin to the Golgi (48,49). Akin the accumulation of endogenous cav-1 in the Golgi of AnxA6-expressing cells, the cavDGV mutant is also retained in the *cis*-Golgi complex. However, further striking similarities can be observed. As shown for AnxA6 in this study, cavDGV causes an intracellular accumulation of cholesterol in LE, a decrease in cholesterol synthesis and cell surface cholesterol, and a reduction in cholesterol efflux (21). Moreover, cavDGV-transfected cells show a significantly reduced number of caveolae. These changes in cavDGV-expressing cells result in dramatic alterations in cholesterol-dependent signaling events, such as the inhibition of the H-Ras-induced activation of Raf-1, and subsequent reduction of growth factor-dependent cell growth (36). Along these lines, we recently showed that AnxA6 inhibits the HDL-induced activation of H-Ras and Raf-1, which requires the binding of HDL to SR-BI in caveolae (23,50). In addition, AnxA6 reduces growth factor-induced Ras/mitogen-activated protein kinase (MAPK) signaling and cell proliferation in CHO and A431 cells (51,52) (T. Grewal, R.J. Daly, C. Enrich, unpublished data). Thus, the mechanisms underlying the effects of cavDGV and AnxA6 on cholesterol and cav-1 transport could provide explanations for the role of caveolae in the regulation of signal transduction and cell growth.

The AnxA6 and cavDGV-expressing cells show a phenotype that partially resembles alterations in cholesterol transport observed in cells carrying a mutation in the NPC1 protein. The NPC1 is involved in cholesterol homeostasis and has been proposed to regulate caveolin expression and localization (20,22). Although the function of the NPC1 protein is not completely understood, NPC1-deficient cells accumulate cholesterol in LE/prelysosomes, delaying cholesterol transport to other caveolin-containing cellular compartments (20,33,37,53) such as the TGN and the PM. In NPC1 ko-cells, the total amount of caveolin at the PM remains unaffected as these cells develop compensatory mechanisms and increase the expression of cav-1 and cav-2 (20). Another member of the NPC1 protein family, NPC1L1, regulates cholesterol absorption at the cell surface of enterocytes. This process appears to involve cav-1 and AnxA2 (54–56), suggesting a co-operativity of some annexins in NPC- and caveolin-dependent cholesterol trafficking events (57). In conclusion, results obtained from studies on NPC1, cav-1 and AnxA6 indicate that the sequestration of cholesterol in the late endocytic compartment and cholesterol synthesis are potentially responsible for subsequent alterations in cav-1 localization/trafficking, cholesterol efflux and signaling events at the PM.

However, whereas caveolin and NPC1/2 are cholesterol-binding proteins and/or directly involved in cholesterol transport, it is believed that annexins, like AnxA6, do not directly interact with cholesterol. Up to date, only AnxA2 has yet been identified in a complex together with cav-1 and cholesterol (56,58). The AnxA6 is thought to predominantly bind to PS (1,58), whereas cholesterol associates preferentially with phosphatidylcholine. Despite these findings, we showed that accumulation of cholesterol stimulates the binding of AnxA6 to LE (14). Similarly, AnxA6 has been shown to relocalize to endocytic compartments in NPC1-deficient cells (59). Furthermore, AnxA6 colocalizes with cav-1 at the PM and in the endocytic compartments has been identified in Triton X-100 (TX-100) insoluble, caveolin- and cholesterol-enriched membrane fractions and translocates to cholesterol-rich membranes in different cell types, including CHO cells (1,17,60) (T. Grewal, K. Gaus, C. Enrich, unpublished data). This indicates that cholesterol modulates the membrane binding and subcellular distribution of AnxA6 (14), which in turn regulates cholesterol and cav-1 transport. Although at present, we cannot completely rule out a subset of AnxA6 proteins being targeted to cholesterol-rich membrane domains at the PM (1,17,60), thereby controlling PM cholesterol, caveolin distribution and caveolae formation, we currently favor several possibilities of AnxA6 acting in LE: AnxA6 could be involved in the formation of PS-rich domains in LE, thereby affecting the activity of proteins residing in neighboring cholesterol-rich domains that control cholesterol transport. Alternatively, the colocalization and co-immunoprecipitation of AnxA6 and NPC1 could indicate that AnxA6 directly downregulates NPC1 activity. This would ultimately control the amount of cav-1 and caveolae at the PM. In support of both models, we showed that the accumulation of Golgi-associated cav-1 in CHOanx6 can be overcome upon addition of exogenous cholesterol or overexpression of NPC1. The fact that AnxA6 is found in the Golgi in some cell types such as hepatocytes and WIF-B cells (6,10) could indicate a direct involvement in cholesterol and cav-1 transport from this compartment. Nevertheless, currently available antibodies did not reveal significant amounts of AnxA6 in the Golgi of CHOanx6 cells.

Recently, overexpression of late endosomal Rab proteins, in particular Rab9 and the recycling/exocytic Rab8, were shown to rescue the cholesterol accumulation in NPC1 mutant cells (42–44). The mechanisms linking NPC1 and Rab proteins are yet unclear, but Rab-dependent membrane transport seems to promote cholesterol export pathways from LE. Interestingly, RNAi experiments revealed that Rab9 is not essential for cholesterol export from LE in normal cells (45). Yet, accumulation of late endosomal cholesterol interferes with the activity cycle of Rab’s (42,45) that requires their release from the membrane into the cytoplasm, and inhibition of Rab’s blocks removal of cholesterol from LE (61). Thus, sequestration of Rab proteins in LE membranes may contribute to the cellular pathology of NPC-related disease. Future studies will have to clarify if high levels of AnxA6, similar to NPC1 mutations, interfere with the trafficking of late endosomal Rab proteins between their target membrane and the cytosol.

### The AnxA6 and the secretory pathway

Data presented in this study suggest that sequestration of cholesterol in LE, possibly together with reduced cholesterol synthesis, in CHOanx6 cells reduces the activity of cholesterol-dependent secretory pathways thereby contributing to an accumulation of cav-1 in the Golgi. This is supported by several studies demonstrating that cholesterol is a limiting factor for the formation of vesicles in the secretory pathway. Both constitutive and regulated formation of secretory vesicles requires cholesterol. Depletion of cholesterol inhibits the formation of secretory vesicles from the TGN (62). Vice versa, increased cellular cholesterol levels induce pronounced vesiculation and dispersal of Golgi-derived vesicles (63). In this fashion, Stüven et al. (38) showed that the cholesterol levels at the Golgi complex must be precisely balanced to allow protein transport to occur.

One candidate protein potentially inhibited by AnxA6 and involved in vesiculation and tubulation events regulating membrane traffic and cargo export from the Golgi could be the cholesterol-dependent cytoplasmic phospholipase A2 (cPLA_2_). The cPLA_2_ translocates to the Golgi apparatus in response to increased cellular cholesterol levels (63), and cPLA_2_ activity is cholesterol sensitive (64). Consistent with this hypothesis, we determined a reduced cPLA_2_ activity in CHOanx6 cells that can be rescued by addition of exogenous cholesterol (unpublished data).

Similarly, the inhibitory effect of other annexins, such as AnxA1, AnxB1 and AnxA5, on cPLA_2_ activity has also been demonstrated (65). This could lead to reduced vesiculation from the Golgi that would downregulate the availability of newly synthesized cholesterol acceptors, such as cav-1, for vesicular or non-vesicular export of cholesterol from LE (66). Besides, annexins have also been related to other aspects of the secretory pathway (1). The AnxA2 is involved in Ca^2+^-regulated exocytosis (4). The AnxA13b associates with sphingolipid and cholesterol-rich domains of the TGN that are required for the budding to the apical PM (67). However, other factors like dynamin (63), the actin cytoskeleton (68) and calmodulin (69) are also involved in this process suggesting that multifactorial protein–lipid interactions facilitate Golgi vesiculation. From these observations, one could envisage that individual annexins modulate various steps of the secretory pathway through different and possibly cholesterol-dependent mechanisms.

The role of AnxA6 in the secretory pathway has not been extensively investigated, and several reports suggested, in agreement with this study, an inhibitory role in the secretory process. First, Zaks and Creutz showed that AnxA6 inhibits vesicle aggregation and fusion, mediated by AnxA2 and AnxA7 (70). Second, AnxA6 is found in non-lactating breast epithelial cells but is undetectable in similar sections taken from lactating breast (71). Finally, hepatocytes expressing high levels of AnxA6 only contain a small number of caveolae (72, 73). As proposed by van Duyl et al. (74), the interaction of caveolin and cholesterol may be fundamental to the generation of caveolae. Defects in the export of caveolin from the Golgi because of the loss of Golgi exit information, exposure of retention signals or conformational changes can contribute to regulate the number of caveolae. Here, we show that alterations in cholesterol transport caused by the expression of AnxA6 result in a slow export of cav-1 from the Golgi. Hence, it is tempting to speculate that this process could include availability of cholesterol for NPC1, cav-1 oligomerization and the delivery of cholesterol to the PM. It is plausible that in certain epithelial cells, including hepatocytes that express abundant amounts of NPC1 (75), and AnxA6 (5,6,10), their interaction contributes to regulate the amount of caveolin/caveolae and cholesterol at the cell surface.

## Materials and Methods

### Reagents and antibodies

Nutrient Mixture Ham’s F-12, DMEM, RPMI-1640, filipin, Cyhx and water-soluble cholesterol, saponin, U18666A and CD were from Sigma. Percoll was from Invitrogen. Protein A/G–Sepharose, [^3^H]-Chol and paraformaldehyde (PFA) was from Electron Microscopy Sciences, and Mowiol was from Calbiochem. Lipoprotein-deficient fetal calf serum (FCS) was prepared by preparative ultracentrifugation as described (76). High-density lipoproteins (HDL_3_, density 1.125–1.21 g/mL) were isolated from the plasma of normolipidemic volunteers by sequential density gradient ultracentrifugation as described (50). After preparation, HDL_3_ was stored in KBr at 4°C and dialyzed extensively against PBS before use. The construction of YFP-caveolin-1 (YFP–cav1), GFP–cav3 and EGFR (EGFR–GFP) expression vectors have been described previously (40,77). The GFP-tagged VSV-G (VSV-G–GFP), Rab7 (Rab7–GFP), MAL (GFP–MAL) and NPC1 (NPC1–GFP) were kindly provided by Dr J. Lippincott-Schwartz (NIH, Bethesda, MD, USA), Dr A. Sorkin (University of Colorado, Denver, CO, USA), Dr M.A. Alonso (Centro de Biologia Molecular ’severo Ochoa’, Universidad Autónoma de Madrid, Madrid, Spain) and Dr P.G. Pentchev (NIH, Bethesda, MD, USA), respectively. For the construction of expression vectors encoding CFP-tagged AnxA6 (Anx6–CFP) and myc-tagged AnxA6 deletion mutant Anx6_1–175_(7) (Anx6_1–175_–myc), rat full length and a 542 bp *Hin*dIII–Asp1 AnxA6 fragment containing coding sequences from pos. +1 to pos. +526 were cloned into pECFP-C1 (Clontech) and pcDNA3.1A-myc-his (Invitrogen), respectively. For the construction of GFP-tagged AnxA6 (GFP–Anx6), a 733 bp EGFP fragment was isolated from pEGFP (BD Transduction Laboratories) and cloned immediately upstream the N-terminus of rat AnxA6 in pcDNAanx6 (8). The RNAi targeting human AnxA6 and GFP were from Santa Cruz Biotechnology. Polyclonal anti-caveolin (C13630), mouse anti-caveolin (PM-associated cav-1) (C43420), mouse anti-GM130, mouse anti-TGN38, mouse anti-GMAP210 were from BD Transduction Laboratories. Polyclonal anti-SR-BI and anti-actin were from Novus and MP Biomedicals, respectively. Monoclonal anti-LBPA was kindly provided by Dr J. Gruenberg (University of Geneva, Geneva, Switzerland). Mouse anti-cav-1 (Golgi-associated cav-1) (03-76000) and horseradish peroxidase (HRP)-conjugated secondary antibodies were from Zymed Laboratories. Mouse anti-myc (9E10), rabbit anti-Rab5, rabbit anti-Rab7 and mouse anti-HRas were from Santa Cruz Biotechnology. Mouse anti-α-1 Na^+^/K^+^ ATPase was from Upstate, mouse anti-KDEL-receptor was from Stressgen Bioreagents, and rabbit anti-GFP was from Abcam. Alexa Fluor-conjugated secondary antibodies and 4,6-diamidino-2-phenylindole dihydrochloride were from Molecular Probes Inc.

### Cell culture

The CHO cells were grown in Ham’s F-12, A431, HeLa and COS-1 cells in DMEM, MDA-MB-468 and MDA-MB-436 in RPMI-1640 together with 10% FCS, L-glutamine (2 mm), penicillin (100 U/mL) and streptomycin (100 μg/mL) at 37°C, 5% CO_2_. To generate stable AnxA6-overexpressing CHO cells, 1 × 10^6^ cells were transfected with 10 μg of pcDNAanx6 and the FUGENE™ 6 Transfection Reagent (Roche Molecular Biochemicals). G418 (1 mg/mL) was added 24 h after transfection. After 2 weeks, G418-resistant colonies were isolated and examined for expression of AnxA6 by Western blotting and immunofluorescence (7,8,14). As described previously (8), CHOanx6 cells showed no obvious morphological alterations of the endocytic compartment (Rab5, EEA1, Rab11 or the recycling of transferrin), lysosomes, Golgi (GM130) or the endoplasmic reticulum by immunofluorescence and electron microscopy (data not shown).

To generate the AnxA6-expressing A431 cell line, A431 cells were transfected with pcDNAanx6 and selected as described above (15). For the transient transfection of expression vectors encoding Anx6, Anx6_1–175_, cav-1, cav-3, Rab7 and NPC1, 1 × 10^5^ CHOwt, CHOanx6 or COS-1 cells were transfected with 1.5 μg Qiagen-purified DNA and 4 μL of Lipofectamine 2000 (Invitrogen) in OptiMEM (Gibco BRL) according to the instructions of the manufacturer. For the intracellular accumulation of cholesterol, cells were treated for 24 h with U18666A (2 μg/mL) as described (14).

### Cholesterol measurements and cyhx/cholesterol treatments

The amount of cholesterol in whole cell lysates (total cholesterol) and PM, EE and LE fractions were determined using the Amplex™ Red Cholesterol Assay Kit (Molecular Probes) as described (16). To determine the amount of cholesterol in PM fractions, 1 × 10^7^ CHOwt and CHOanx6 cells were washed twice in 0.25 m sucrose, 1 mm ethylenediaminetetraacetic acid (EDTA), 20 mm Tris–HCL, pH 7.8 plus protease inhibitors, collected, lysed and centrifuged. The postnuclear supernatant (PNS) was layered on top of 10 mL of 30% Percoll and centrifuged at 84 000 ×***g***for 30 min in a Beckman 70.1 TI rotor as described (15). Fractions (1 mL) were taken from top to bottom, and the amount of cholesterol in 50 μL of each fraction was determined and normalized to total protein (78). To identify the PM-containing fractions in the middle of the gradient, aliquots of each fraction were analyzed for the distribution of Ras by Western blotting (15) (see also Figure S1B).

For the determination of cholesterol in LE and EE, 4–6 × 10^7^ CHOwt and CHOanx6 cells were homogenized by 10 passages through a 22-gauge needle, and endosomes were separated on sucrose gradients as described (8,14). In brief, the cell homogenate was centrifuged, and the PNS was brought to a final 42% sucrose (w/v) concentration. Then 35% sucrose, 25% sucrose and homogenization buffer were poured stepwise on top of the PNS. The gradient was centrifuged for 90 min at 155 000 ×***g***. After centrifugation, 1 mL fractions were collected from top to bottom, and fractions representing EE and LE were pooled. Before further analysis, the purity of fractions was routinely confirmed by Western blot analysis with PM, EE and LE markers and β-hexosaminidase assays (8) (see also Figure S1A). For the fluorometric quantification of cholesterol, 25 μL of LE and EE together with cholesterol standards (Precinorm™, Precipath™; Roche Molecular Biochemicals) were incubated in 24-well plates according to the instructions of the manufacturer, and fluorescence was detected using Fluorocount™ (Packard Instrument Co.).

To determine cholesterol levels in the Golgi apparatus, a modified method of Balch and coworkers (79) described in detail by Brügger et al. (80) was used to isolate Golgi membrane fractions from CHO cells. All procedures were carried out at 4°C. In brief, 4–5 × 10^9^ cells were harvested, washed twice with PBS, twice with breaking buffer (BB) (0.25 m sucrose in 10 mm Tris–HCl, pH 7.4) and resuspended in four volumes of BB. Cells were homogenized by passing 25 times through a ball-bearing homogenizer (Balch homogenizer), which disrupts EE and LE, but not Golgi vesicles, and brought to a sucrose concentration of 37% (w/w) by addition of 62% (w/w) sucrose. Fourteen milliliters of sample was overlaid with 15 mL 35% (w/w) sucrose and 9 mL 29% (w/w) sucrose (in 10 mm Tris–HCl, pH 7.4) and centrifuged for 2.5 h at 82 700 ×***g***. Typically, 2 mL of a Golgi-enriched membrane fraction was recovered at the 35–29% interphase. The purity of the isolated Golgi membranes was confirmed by Western blot analysis with Golgi (TGN38, GM130 and GMAP210), EE (Rab5), LE (Rab7), PM (Na^+^K^+^ ATPase) and endoplasmic reticulum (KDEL) markers (Figure S1C). To determine cholesterol levels, 500 μg of purified Golgi fraction were mixed with 0.3 mL KOH (33%), 0.3 mL of ethanol (95%) and incubated at 60°C for 15 min as described (81). After addition of 10 mL of hexane and 3 mL of distilled water, samples were mixed, and 4 mL of the organic hexane phase were, together with cholesterol standard (0–60 mg in ethanol), evaporated under nitrogen. Two milliliters of freshly prepared ophtaladehyde solution (0.5 mg/mL in glacial acetic acid) and after 5 min, 1 mL of concentrated sulfuric acid, were added. After 10 min, the absorbance was read at 550 nm.

For the determination of cholesterol synthesis, 4 × 10^5^ CHOwt and CHOanx6 cells (in triplicate) were grown in media containing 5% lipoprotein-deficient FCS and incubated overnight with 1 μCi/mL [1-^14^C]-acetic acid as described in Du et al. (35). Cells were washed twice in buffer C (5 mm Tris–HCL, pH 7.5; 150 mm NaCl) and 0.2% BSA, twice in buffer C alone and lysed in 1 mL of 0.1 m NaOH. Then cells were saponified with 1 mL of 75% (w/v) KOH, 2 mL ethanol, 20 μm butylated-hydroxytoluene, 20 μm EDTA at 70°C for 1 h. After cooling, nonsaponifiable lipids were extracted into hexane (3 × 3 mL) and evaporated to dryness. Lipid extracts were redissolved in 100 μL hexane and separated by TLC using 4% (w/v) silver-coated Silica Gel 60 F_254_ plates (Merck) and a mobile phase of heptane:ethyl acetate (2:1, v/v). This method separates cholesterol from its major precursors including desmosterol. For visualization and quantification, TLC plates were exposed to X-ray film, quantified using ImageJ (NIH) and normalized for total cellular protein (78).

To inhibit protein synthesis in some experiments, 10 μg/mL Cyhx (in 100 mm Hepes, pH 7.5) was added to the media for 90 min. For cholesterol addition, cells were incubated with 30 μg/mL cholesterol and premixed at room temperature for 30 min in DMEM by gentle agitation. When Cyhx and cholesterol were used in combination, both were dissolved in DMEM. The Cyhx completely inhibited protein synthesis as judged by the lack of any detectable fluorescence after expression of GFP in the presence of Cyhx for 24 h (unpublished data).

### The RNAi-mediated inhibition of AnxA6

HeLa cells (1–2 × 10^6^) were transfected in 2 mL medium with 10 nm AnxA6 small interfering RNA (siRNA) and 6 μL of Lipofectamine 2000 reagent (Invitrogen) according to the instructions of the manufacturer as described (15). Experiments were conducted 72 h after transfection. The GFP siRNA was used as a negative control.

### Efflux experiments

To measure cholesterol efflux from LE, cells were treated for 24 h ± U18666A (2 μg/mL) in the presence of [^3^H]-Chol (2 × 10^6^ c.p.m/mL). To remove non-internalized [^3^H]-Chol, cells were washed extensively, followed by incubation in serum-free media ± 1% CD for 120 min. Then, the radioactivity in the media and cell lysate was collected, determined by scintillation counting (82) and normalized to total cellular protein (78). The ratio of released and cell-associated radioactivity was determined and is given in per cent. The background efflux obtained from U18666A-treated CHOwt and CHOanx6 cells was 0.8–1.2% (equivalent to 5.0–7.0 × 10^5^ c.p.m/mg cell protein), respectively.

For the determination of HDL_3_-induced cholesterol efflux, cells were labeled with radioactive LDL. To label LDL with [^3^H]-Chol, 100 μL of [^3^H]-Chol (3.7 Mbq) was dried under liquid nitrogen, resuspended in 100 μL of DMEM + 2% BSA and incubated overnight with 3–5 mg LDL in PBS and 10 mm EDTA at 37°C. Non-incorporated [^3^H]-Chol was removed by PD10 gel chromatography (Amersham Biosciences). Then 2–5 × 10^5^ cells (in triplicate) were incubated overnight with [^3^H]-Chol–LDL (1 × 10^6^ c.p.m/mL), and non-internalized radioactivity was removed by extensive washing with PBS. To determine HDL_3_-induced cholesterol efflux, cells were incubated in Ham’s F12/0.1% BSA ± 5, 50 or 100 μg/mL HDL_3_ for 8 h. The media were harvested, cells were lysed in 0.1 N NaOH, and the total cellular protein was determined (78). The radioactivity in the media and cell lysate was determined by scintillation counting (82). The ratio of released and cell-associated radioactivity was determined and is given in per cent as above.

### Immunofluorescence

Cells were grown on coverslips and fixed with 4% PFA, washed, permeabilized with 0.1% saponin and incubated with primary and secondary antibodies or filipin (5 μg/mL) as described elsewhere (21). At last, samples were mounted in Mowiol, and the cells were observed using an oil-immersion Plan-Apo63x/1.4 objective in an Axio-plan or Axiovert 200M Zeiss microscope (Zeiss). In the Axio-plan microscope, the images were captured with an AxioCam HRc camera and were digitally treated with AxioVision 3.1 software. In the 200M microscope, the images were captured with a Photometrics Cool Snap HQ camera controlled by slide-book 3.0.10.5 software (Intelligent Imaging Innovation). Image analysis was performed with Adobe-Photoshop 7.0 software. To quantify the fluorescence intensity of cav-1 or cholesterol (filipin) staining, experiments were performed in parallel, and images were captured using identical contrast and exposure times. Utilizing NIH image (ImageJ), the area to be quantified (e.g. Golgi for cav-1) was selected, the pixel intensity was determined, and the average staining intensity (50–150 cells per cell line in –three to five experiments, respectively) was calculated. For the quantification and size determination (nm Ø) of LBPA-positive vesicles, 100 cells per cell line per treatment in three independent experiments were analyzed.

Different experimental set-ups were used for the imaging of CFP- and GFP-cotransfected cells and for filipin in GFP-transfected cells (see below). For the imaging of CFP–Anx6 and GFP–Rab7 in COS-1 cells, a Leica TCS-SL spectral confocal microscope with 63× oil immersion objective lens (NA 1.32) was used. To resolve these two fluorophores, the following configuration was used in a sequential mode (for CFP, 458 nm laser line; excitation beam splitter double dichroic 458/514 nm, emission detection range 465–485 nm; for GFP, 488 nm laser line; excitation beam splitter triple dichroic 488/543/633 nm, emission detection range 500–550 nm).

Images of filipin and GFP-labeled cells were acquired using a Leica DMI6000B epifluorescence microscope equipped with a Leica DFC 350 FX monochroma camera and 100× oil immersion objective lens (NA 1.4). Filipin was imaged using a 360-nm filter cube (40-nm bandpass) excitation filter (cross-excitation of GFP is null or unappreciated), 400-nm longpass dichromatic filter and 425-nm (longpass) emission filter. The GFP was imaged using a 480-nm filter cube (40-nm bandpass) excitation filter (filipin cannot be excited by 460–500 nm), 505-nm longpass dichromatic filter and 527-nm (30-nm bandpass) emission filter. This, together with the sequential acquisition of images, made it possible to discard fluorescence bleed through or cross excitation (measured using single labeled samples).

### Photobleaching experiments and time-lapse confocal microscopy

The iFRAP experiments were carried out using a Leica TCS SL laser scanning confocal spectral microscope (Leica Microsystems) with Argon and HeNe lasers attached to a Leica DMIRE2 inverted microscope equipped with an incubation system with temperature and CO_2_ control. For visualization of GFP, images were acquired using 63× oil immersion objective lens (NA 1.32; 488 nm laser line; excitation beam splitter RSP 500, emission range detection 500–600 nm). The confocal pinhole was set at 2–3 Airy units to minimize changes in fluorescence because of GFP-tagged proteins moving away from the plane of focus. The whole cytoplasm, except the Golgi of a GFP-fusion protein-transfected cell, was photobleached using 50–80 scans with the 488 nm laser line at full power. Prebleach and postbleach images were monitored at 30-second intervals for 90 min. The excitation intensity was attenuated down to approximately 5% of the half laser power to avoid significant photobleaching. The relative loss of fluorescence intensity in the unbleached region of interest and overall photobleaching in the whole cell during the time series were quantified using Image Processing Leica Confocal Software. Background fluorescence was measured in a random field outside of the cells. Fluorescence correction, normalization and kinetic parameters (mobile fraction and half time of equilibration) of GFP-tagged proteins were calculated according to Rabut and Ellenberg (83).

### Immunoprecipitation and Western blotting

For the immunoprecipitation of Golgi-associated cav-1, the method previously described in Pol et al. (40) was used and 1 × 10^7^ CHOwt, CHOanx6, A431wt and HeLa cells were extracted with 1 mL of ice-cold 10 mm Tris, pH 7.5, 150 mm NaCl, 5 mm EDTA and protease and phosphatase inhibitors, supplemented with 0.1% TX-100. After removal of cell debris, the amount of extracted protein was quantified (78). Two micrograms of anti-caveolin (Golgi-associated cav-1) was mixed with 120 μg of sample for 2 h at 4°C and antibody/caveolin complexes were collected with protein A/G–Sepharose and separated on 12% SDS–PAGE (see below).

For the co-immunoprecipitation of AnxA6 and NPC1, CHOanx6 were transfected with GFP–NPC1, and immunoprecipitation of AnxA6 was performed as described (15). Cells were washed twice in 5 mL of homogenization buffer (HB) (50 mm Tris, 150 mm NaCl and 5 mm EDTA, pH 8.0) and scraped in 1 mL of lysis buffer (HB plus 5 mm NaF, 0.2 mm Na_2_VO_3_, 0.1% (v/v) Triton-X-100 and protease inhibitors). The protein content was determined, and then 500–800 μg of cell lysate was incubated with 2 μg of anti-AnxA6 (rabbit) and control antibodies for 120 min at 4°C. Then 6 μL of Protein G Sepharose (Pharmacia) was added, and samples were incubated for 45 min at 4°C, centrifuged and washed three times in buffer HB. The immunoprecipitates were pelleted, washed and separated by 12% SDS–PAGE.

After transfer to Immobilon membranes, AnxA6, cav-1 and NPC1 were detected using polyclonal antibodies, respectively, followed by HRP-conjugated secondary antibodies and enhanced chemiluminescence detection (Amersham).

### Electron microscopy

Cell monolayers on 10-cm dishes were rinsed with PBS and fixed with 2% PFA and 2.5% glutaraldehyde in 0.1 m phosphate buffer for 1 h at room temperature. Cells were gently scraped, collected and pelleted by centrifugation (5 min at 500 ×***g***). After three rinses in 0.1 m phosphate buffer, pellets were postfixed in 1% OsO_4_–0.8% FeCNK for 90 min. Finally, samples were embedded in Spurr (Sigma), and ultrathin sections were analyzed with a Jeol1010 electron microscope. Grids were systematically screened, and the number of caveolae (non-coated 50–80 nm surface flask-shaped membrane invaginations) were determined in random fields of sections from images acquired at the same magnification (25 000×). For each cell line and condition, over 50 cells were examined with an estimated total lineal surface of 500 μm. Similar results were obtained in three independent experiments.

## Supplementary Materials

**Supplemental Figure 1: (A-C) Western blot analysis of Sucrose gradients (A), Percoll gradients (B) and Golgi membrane fractions (C) from CHOwt cells.** (A) Early (EE) and late (LE) endosomal membrane fractions (20 μg protein/fraction) are characterized by the presence of Rab5 and Rab7 and are well separated from the bulk membrane (Na^+^/K^+^ ATPase) at the bottom of the gradient (8, 16). (B) Plasma membrane (PM) enriched membrane fractions (20 μg protein/fraction) are characterized by the enrichment of Ras (15). Incubation of cells overnight with ^3^H-Cholesterol predominantly labels the Ras-containing fractions 4 and 5. (C) Analysis of Golgi membrane fractions (Golgi, 100 μg protein) and whole cell lysates (WCL, 100 μg protein) shows the enrichment of Golgi markers GM130, TGN38 and GMAP210 (79, 80). No significant amounts of EE, LE and PM markers (Rab5, Rab7, KDEL, Na^+^/K^+^ ATPase) were detected. Gradients from CHOanx6 cells showed similarly well separated endosomal, PM and Golgi membrane fractions (not shown).

**Supplemental Figure 2: Cholesterol efflux is decreased in AnxA6 expressing cells.** (A) CHOwt and CHOanx6 were incubated with [^3^H]-Chol (2 × 10^6^ cpm/ml) for 24 h. Cells were washed with PBS and incubated ± 1 % methyl-β-cyclodextrin (CD) for 120 min. The ratio of released and cell-associated radioactivity was determined, normalized to total cell protein and the amount of efflux is given in (%). The background efflux obtained from CHOwt and CHOanx6 was equivalent to 2.0 − 8.0 × 10^5^ cpm/mg cell protein, respectively. Mean values ± S.D. of 3 independent experiments with triplicate samples are given. *, p<0.05 for student's *t* test. Alternatively, CHOwt and CHOanx6 cells were labeled overnight with [^3^H]-Chol-LDL (1 × 10^6^ cpm/ml) and efflux was induced with 5 – 100 μg/ml HDL3 for 8 h as indicated. The percentage of [^3^H]-Chol efflux was calculated as described above and represents the mean ± S.D. of three independent experiments with triplicate samples. (B) A431wt, A431anx6, BT20wt (express low amounts of AnxA6, unpublished data) and BT20anx6 were incubated with 2 μg/ml U18666A together with [^3^H]-Chol (2 × 10^6^ cpm/ml cpm/ml) for 24 h. Cells were washed with PBS and incubated ± 1 % methyl-β-cyclodextrin (CD) for 120 min. The ratio of released and cell-associated radioactivity was determined, normalized to total cell protein and the amount of efflux is given in (%). The background efflux in U18666A-treated cells was equivalent to 3.0 – 6.0 × 10^5^ cpm/mg cell protein), respectively. Mean values ± S.D. of 3 independent experiments with triplicate samples are given. **, p<0.01 for student's t test. (C) 4 × 10^5^ CHOwt and CHOanx6 cells (in triplicate) were grown in media containing 5 % lipoprotein-deficient FCS and incubated overnight with 1 μCi/ml [1-^14^C]-acetic acid. Lipids were extracted and newly synthesized cholesterol was separated from its major precursors including desmosterol by thin layer chromatography (TLC) as described (35). For visualization and quantification, TLC plates were exposed to X-ray film, and the amount of radioactive cholesterol was quantified using ImageJ (NIH). Relative intensities were normalized for total cellular protein (78). The mean values for a representative experiment are given. In 2 independent experiments with triplicate samples an overall reduction of cholesterol synthesis in CHOanx6 cells by 36 ± 18 % (p<0.05) was observed.

**Supplemental Figure 3: Caveolin accumulates in a perinuclear compartment of AnxA6 expressing cells.** (A) CHOwt (a) and CHOanx6 (b) cells grown on coverslips were fixed, permeabilized and stained with polyclonal anti-caveolin. Arrowheads point at the perinuclear accumulation of caveolin in CHOanx6 cells. Images are representative for 90% of cells analyzed. The fluorescence intensity of the perinuclear cav-1 staining in CHOwt and CHOanx6 cells was quantified. Values represent the mean ± S.D. of 5 independent experiments with 50 cells per cell line in each experiment. ***, p<0.001 for student's *t* test. (B) CHO wildtype cells (a,b) were transiently transfected with GFP-Anx6 and stained with polyclonal anti-caveolin. Arrowheads point at the accumulation of cav-1 in the perinuclear Golgi region of the transfected cell. The protein levels of cav-1, GFP-Anx6 and endogenous AnxA6 in transfected (+) and non-transfected (-) CHOwt cells are given. (C) Western blot analysis of cav-1, Scavenger receptor BI (SR-BI), H-Ras and AnxA6 from total cell lysates of CHOwt and CHOanx6 cells.

**Supplemental Figure 4: Cholesterol restores the cellular distribution of cav-1 in CHOanx6 cells.** CHOwt (a, c) and CHOanx6 cells (b, d) were grown on coverslips, incubated ± cholesterol for 90 min, fixed, permeabilized and immunolabeled with anti-caveolin as indicated. Images are representative for 3 independent experiments. Arrows point at the localization of endogenous cav-1 at the PM in CHOwt and CHOanx6 cells upon addition of cholesterol (c and d). Bar is 10 μm. (E) CHOwt (lane 1 – 4) and CHOanx6 cells (lane 5 – 8) were incubated with cholesterol and Cyhx for 90 min as indicated. Then the Golgi-associated cav-1 was immunoprecipitated and analyzed by Western blotting as described (40). The amount of immunoprecipitated cav-1 from the Golgi was quantified and normalized to actin. The relative amount of cav-1 in the Golgi is given and is representative for 3 independent experiments. ***, p<0.001 for student's *t* test.

**Supplemental Figure 5: Cholesterol restores the ability of CHOanx6 cells to re-establish cholesterol sorting events of VSV-G.** CHOwt (a, c) and CHOanx6 cells (b, d) were grown on coverslips and transfected with VSVG-GFP. 24 h after transfection, cells were incubated ± cholesterol for 90 min, fixed, permeabilized and analyzed by epifluorescence microscopy. The arrow points at the perinuclear localization of GFP-tagged VSV-G in CHOanx6 cells upon addition of cholesterol (d). Results are representative for 3 independent experiments. Bar is 10 μm.

## References

[1] Gerke V, Creutz CE, Moss SE (2005). Annexins: linking Ca2+ signalling to membrane dynamics. Nat Rev Mol Cell Biol.

[2] Grewal T, Enrich C (2006). Molecular mechanisms involved in Ras inactivation: the annexin A6-p120GAP complex. Bioessays.

[3] Raynal P, Pollard HB (1994). Annexins: the problem of assessing the biological role for a gene family of multifunctional calcium- and phospholipid-binding proteins. Biochim Biophys Acta.

[4] Gerke V, Moss SE (2002). Annexins: from structure to function. Physiol Rev.

[5] Ortega D, Pol A, Biermer M, Jackle S, Enrich C (1998). Annexin VI defines an apical endocytic compartment in rat liver hepatocytes. J Cell Sci.

[6] Jackle S, Beisiegel U, Rinninger F, Buck F, Grigoleit A, Block A, Groger I, Greten H, Windler E (1994). Annexin VI, a marker protein of hepatocytic endosomes. J Biol Chem.

[7] Pons M, Grewal T, Rius E, Schnitgerhans T, Jackle S, Enrich C (2001). Evidence for the involvement of annexin 6 in the trafficking between the endocytic compartment and lysosomes. Exp Cell Res.

[8] Grewal T, Heeren J, Mewawala D, Schnitgerhans T, Wendt D, Salomon G, Enrich C, Beisiegel U, Jackle S (2000). Annexin VI stimulates endocytosis and is involved in the trafficking of low density lipoprotein to the prelysosomal compartment. J Biol Chem.

[9] Kamal A, Ying Y, Anderson RG (1998). Annexin VI-mediated loss of spectrin during coated pit budding is coupled to delivery of LDL to lysosomes. J Cell Biol.

[10] Pons M, Ihrke G, Koch S, Biermer M, Pol A, Grewal T, Jackle S, Enrich C (2000). Late endocytic compartments are major sites of annexin VI localization in NRK fibroblasts and polarized WIF-B hepatoma cells. Exp Cell Res.

[11] Pol A, Ortega D, Enrich C (1997). Identification of cytoskeleton-associated proteins in isolated rat liver endosomes. Biochem J.

[12] Seemann J, Weber K, Osborn M, Parton RG, Gerke V (1996). The association of annexin I with early endosomes is regulated by Ca2+ and requires an intact N-terminal domain. Mol Biol Cell.

[13] Desjardins M, Celis JE, van Meer G, Dieplinger H, Jahraus A, Griffiths G, Huber LA (1994). Molecular characterization of phagosomes. J Biol Chem.

[14] de Diego I, Schwartz F, Siegfried H, Dauterstedt P, Heeren J, Beisiegel U, Enrich C, Grewal T (2002). Cholesterol modulates the membrane binding and intracellular distribution of annexin 6. J Biol Chem.

[15] Grewal T, Evans R, Rentero C, Tebar F, Cubells L, de Diego I, Kirchhoff MF, Hughes WE, Heeren J, Rye KA, Rinninger F, Daly RJ, Pol A, Enrich C (2005). Annexin A6 stimulates the membrane recruitment of p120GAP to modulate Ras and Raf-1 activity. Oncogene.

[16] Massey-Harroche D, Mayran N, Maroux S (1998). Polarized localizations of annexins I, II, VI and XIII in epithelial cells of intestinal, hepatic and pancreatic tissues. J Cell Sci.

[17] Babiychuk EB, Draeger A (2000). Annexins in cell membrane dynamics. Ca(2+)-regulated association of lipid microdomains. J Cell Biol.

[18] Smythe E, Smith PD, Jacob SM, Theobald J, Moss SE (1994). Endocytosis occurs independently of annexin VI in human A431 cells. J Cell Biol.

[19] Hawkins TE, Roes J, Rees D, Monkhouse J, Moss SE (1999). Immunological development and cardiovascular function are normal in annexin VI null mutant mice. Mol Cell Biol.

[20] Garver WS, Krishnan K, Gallagos JR, Michikawa M, Francis GA, Heidenreich RA (2002). Niemann-Pick C1 protein regulates cholesterol transport to the trans-Golgi network and plasma membrane caveolae. J Lipid Res.

[21] Pol A, Luetterforst R, Lindsay M, Heino S, Ikonen E, Parton RG (2001). A caveolin dominant negative mutant associates with lipid bodies and induces intracellular cholesterol imbalance. J Cell Biol.

[22] Ikonen E, Parton RG (2000). Caveolins and cellular cholesterol balance. Traffic.

[23] Rentero C, Evans R, Wood P, Tebar F, Vila de Muga S, Cubells L, de Diego I, Hayes TE, Hughes WE, Pol A, Rye KA, Enrich C, Grewal T (2006). Inhibition of H-Ras and MAPK is compensated by PKC-dependent pathways in annexin A6 expressing cells. Cell Signal.

[24] Stenmark H, Parton RG, Steele-Mortimer O, Lutcke A, Gruenberg J, Zerial M (1994). Inhibition of rab5 GTPase activity stimulates membrane fusion in endocytosis. EMBO J.

[25] Bucci C, Parton RG, Mather IH, Stunnenberg H, Simons K, Hoflack B, Zerial M (1992). The small GTPase rab5 functions as a regulatory factor in the early endocytic pathway. Cell.

[26] Sokol J, Blanchette-Mackie J, Kruth HS, Dwyer NK, Amende LM, Butler JD, Robinson E, Patel S, Brady RO, Comly ME (1988). Type C Niemann-Pick disease. Lysosomal accumulation and defective intracellular mobilization of low density lipoprotein cholesterol. J Biol Chem.

[27] Frolov A, Zielinski SE, Crowley JR, Dudley-Rucker N, Schaffer JE, Ory DS (2003). NPC1 and NPC2 regulate cellular cholesterol homeostasis through generation of low density lipoprotein cholesterol-derived oxysterols. J Biol Chem.

[28] Puri V, Jefferson JR, Singh RD, Wheatley CL, Marks DL, Pagano RE (2003). Sphingolipid storage induces accumulation of intracellular cholesterol by stimulating SREBP-1 cleavage. J Biol Chem.

[29] Narita K, Choudhury A, Dobrenis K, Sharma DK, Holicky EL, Marks DL, Walkley SU, Pagano RE (2005). Protein transduction of Rab9 in Niemann-Pick C cells reduces cholesterol storage. Faseb J.

[30] Mobius W, van Donselaar E, Ohno-Iwashita Y, Shimada Y, Heijnen HF, Slot JW, Geuze HJ (2003). Recycling compartments and the internal vesicles of multivesicular bodies harbor most of the cholesterol found in the endocytic pathway. Traffic.

[31] Goldstein JL, Brown MS (1992). Lipoprotein receptors and the control of plasma LDL cholesterol levels. Eur Heart J.

[32] Brown MS, Goldstein JL (1999). A proteolytic pathway that controls the cholesterol content of membranes, cells, and blood. Proc Natl Acad Sci U S A.

[33] Wojtanik KM, Liscum L (2003). The transport of low density lipoprotein-derived cholesterol to the plasma membrane is defective in NPC1 cells. J Biol Chem.

[34] Neufeld EB, Cooney AM, Pitha J, Dawidowicz EA, Dwyer NK, Pentchev PG, Blanchette-Mackie EJ (1996). Intracellular trafficking of cholesterol monitored with a cyclodextrin. J Biol Chem.

[35] Du X, Kristiana I, Wong J, Brown AJ (2006). Involvement of Akt in ER-to-Golgi transport of SCAP/SREBP: a link between a key cell proliferative pathway and membrane synthesis. Mol Biol Cell.

[36] Roy S, Luetterforst R, Harding A, Apolloni A, Etheridge M, Stang E, Rolls B, Hancock JF, Parton RG (1999). Dominant-negative caveolin inhibits H-Ras function by disrupting cholesterol-rich plasma membrane domains. Nat Cell Biol.

[37] Ikonen E (2006). Mechanisms for cellular cholesterol transport: defects and human disease. Physiol Rev.

[38] Stuven E, Porat A, Shimron F, Fass E, Kaloyanova D, Brugger B, Wieland FT, Elazar Z, Helms JB (2003). Intra-Golgi protein transport depends on a cholesterol balance in the lipid membrane. J Biol Chem.

[39] Sandhoff R, Brugger B, Jeckel D, Lehmann WD, Wieland FT (1999). Determination of cholesterol at the low picomole level by nano-electrospray ionization tandem mass spectrometry. J Lipid Res.

[40] Pol A, Martin S, Fernandez MA, Ingelmo-Torres M, Ferguson C, Enrich C, Parton RG (2005). Cholesterol and fatty acids regulate dynamic caveolin trafficking through the Golgi complex and between the cell surface and lipid bodies. Mol Biol Cell.

[41] Lebrand C, Corti M, Goodson H, Cosson P, Cavalli V, Mayran N, Faure J, Gruenberg J (2002). Late endosome motility depends on lipids via the small GTPase Rab7. EMBO J.

[42] Linder MD, Uronen RL, Holtta-Vuori M, van der Sluijs P, Peranen J, Ikonen E (2007). Rab8-dependent recycling promotes endosomal cholesterol removal in normal and sphingolipidosis cells. Mol Biol Cell.

[43] Walter M, Davies JP, Ioannou YA (2003). Telomerase immortalization upregulates Rab9 expression and restores LDL cholesterol egress from Niemann-Pick C1 late endosomes. J Lipid Res.

[44] Choudhury A, Dominguez M, Puri V, Sharma DK, Narita K, Wheatley CL, Marks DL, Pagano RE (2002). Rab proteins mediate Golgi transport of caveola-internalized glycosphingolipids and correct lipid trafficking in Niemann-Pick C cells. J Clin Invest.

[45] Ganley IG, Pfeffer SR (2006). Cholesterol accumulation sequesters Rab9 and disrupts late endosome function in NPC1-deficient cells. J Biol Chem.

[46] Ying M, Grimmer S, Iversen TG, Van Deurs B, Sandvig K (2003). Cholesterol loading induces a block in the exit of VSVG from the TGN. Traffic.

[47] Fu Y, Hoang A, Escher G, Parton RG, Krozowski Z, Sviridov D (2004). Expression of caveolin-1 enhances cholesterol efflux in hepatic cells. J Biol Chem.

[48] Ren X, Ostermeyer AG, Ramcharan LT, Zeng Y, Lublin DM, Brown DA (2004). Conformational defects slow Golgi exit, block oligomerization, and reduce raft affinity of caveolin-1 mutant proteins. Mol Biol Cell.

[49] Luetterforst R, Stang E, Zorzi N, Carozzi A, Way M, Parton RG (1999). Molecular characterization of caveolin association with the Golgi complex: identification of a cis-Golgi targeting domain in the caveolin molecule. J Cell Biol.

[50] Grewal T, de Diego I, Kirchhoff MF, Tebar F, Heeren J, Rinninger F, Enrich C (2003). High density lipoprotein-induced signaling of the MAPK pathway involves scavenger receptor type BI-mediated activation of Ras. J Biol Chem.

[51] Theobald J, Hanby A, Patel K, Moss SE (1995). Annexin VI has tumour-suppressor activity in human A431 squamous epithelial carcinoma cells. Br J Cancer.

[52] Theobald J, Smith PD, Jacob SM, Moss SE (1994). Expression of annexin VI in A431 carcinoma cells suppresses proliferation: a possible role for annexin VI in cell growth regulation. Biochim Biophys Acta.

[53] Ikonen E, Holtta-Vuori M (2004). Cellular pathology of Niemann-Pick type C disease. Semin Cell Dev Biol.

[54] Valasek MA, Weng J, Shaul PW, Anderson RG, Repa JJ (2005). Caveolin-1 is not required for murine intestinal cholesterol transport. J Biol Chem.

[55] Davies JP, Scott C, Oishi K, Liapis A, Ioannou YA (2005). Inactivation of NPC1L1 causes multiple lipid transport defects and protects against diet-induced hypercholesterolemia. J Biol Chem.

[56] Smart EJ, De Rose RA, Farber SA (2004). Annexin 2-caveolin 1 complex is a target of ezetimibe and regulates intestinal cholesterol transport. Proc Natl Acad Sci U S A.

[57] Strzelecka-Kiliszek A, Tylki-Szymanska A, Bandorowicz-Pikula J (2004). Annexins in Niemann-Pick type C disease. Annexins.

[58] Ayala-Sanmartin J (2001). Cholesterol enhances phospholipid binding and aggregation of annexins by their core domain. Biochem Biophys Res Commun.

[59] te Vruchte D, Lloyd-Evans E, Veldman RJ, Neville DC, Dwek RA, Platt FM, van Blitterswijk WJ, Sillence DJ (2004). Accumulation of glycosphingolipids in Niemann-Pick C disease disrupts endosomal transport. J Biol Chem.

[60] Orito A, Kumanogoh H, Yasaka K, Sokawa J, Hidaka H, Sokawa Y, Maekawa S (2001). Calcium-dependent association of annexin VI, protein kinase C alpha, and neurocalcin alpha on the raft fraction derived from the synaptic plasma membrane of rat brain. J Neurosci Res.

[61] Holtta-Vuori M, Maatta J, Ullrich O, Kuismanen E, Ikonen E (2000). Mobilization of late-endosomal cholesterol is inhibited by Rab guanine nucleotide dissociation inhibitor. Curr Biol.

[62] Wang Y, Thiele C, Huttner WB (2000). Cholesterol is required for the formation of regulated and constitutive secretory vesicles from the trans-Golgi network. Traffic.

[63] Grimmer S, Ying M, Walchli S, van Deurs B, Sandvig K (2005). Golgi vesiculation induced by cholesterol occurs by a dynamin- and cPLA2-dependent mechanism. Traffic.

[64] Klapisz E, Masliah J, Bereziat G, Wolf C, Koumanov KS (2000). Sphingolipids and cholesterol modulate membrane susceptibility to cytosolic phospholipase A(2). J Lipid Res.

[65] Kim SW, Rhee HJ, Ko J, Kim YJ, Kim HG, Yang JM, Choi EC, Na DS (2001). Inhibition of cytosolic phospholipase A2 by annexin I. Specific interaction model and mapping of the interaction site. J Biol Chem.

[66] Maxfield FR, Wustner D (2002). Intracellular cholesterol transport. J Clin Invest.

[67] Lafont F, Lecat S, Verkade P, Simons K (1998). Annexin XIIIb associates with lipid microdomains to function in apical delivery. J Cell Biol.

[68] Egea G, Lazaro-Dieguez F, Vilella M (2006). Actin dynamics at the Golgi complex in mammalian cells. Curr Opin Cell Biol.

[69] Tebar F, Villalonga P, Sorkina T, Agell N, Sorkin A, Enrich C (2002). Calmodulin regulates intracellular trafficking of epidermal growth factor receptor and the MAPK signaling pathway. Mol Biol Cell.

[70] Zaks WJ, Creutz CE (1990). Annexin-chromaffin granule membrane interactions: a comparative study of synexin, p32 and p67. Biochim Biophys Acta.

[71] Donnelly SR, Moss SE (1997). Annexins in the secretory pathway. Cell Mol Life Sci.

[72] Everson WV, Smart EJ, Fielding CJ (2006). Caveolin and its role in intracellular chaperone complexes. Lipid Rafts and Caveolae.

[73] Calvo M, Tebar F, Lopez-Iglesias C, Enrich C (2001). Morphologic and functional characterization of caveolae in rat liver hepatocytes. Hepatology.

[74] van Duyl BY, Meeldijk H, Verkleij AJ, Rijkers DT, Chupin V, de Kruijff B, Killian JA (2005). A synergistic effect between cholesterol and tryptophan-flanked transmembrane helices modulates membrane curvature. Biochemistry.

[75] Garver WS, Xie C, Repa JJ, Turley SD, Dietschy JM (2005). Niemann-Pick C1 expression is not regulated by the amount of cholesterol flowing through cells in the mouse. J Lipid Res.

[76] Goldstein JL, Basu SK, Brown MS (1983). Receptor-mediated endocytosis of low-density lipoprotein in cultured cells. Methods Enzymol.

[77] Llado A, Tebar F, Calvo M, Moreto J, Sorkin A, Enrich C (2004). Protein kinaseCdelta-calmodulin crosstalk regulates epidermal growth factor receptor exit from early endosomes. Mol Biol Cell.

[78] Lowry OH, Rosebrough NJ, Farr AL, Randall RJ (1951). Protein measurement with the Folin phenol reagent. J Biol Chem.

[79] Balch WE, Dunphy WG, Braell WA, Rothman JE (1984). Reconstitution of the transport of protein between successive compartments of the Golgi measured by the coupled incorporation of N-acetylglucosamine. Cell.

[80] Brügger B, Sandhoff R, Wegehingel S, Gorgas K, Malsam J, Helms JB, Lehmann WD, Nickel W, Wieland FT (2000). Evidence for segregation of sphingomyelin and cholesterol during formation of COPI-coated vesicles. J Cell Biol.

[81] Rudel LL, Morris MD (1973). Determination of cholesterol using o-phthalaldehyde. J Lipid Res.

[82] Heeren J, Grewal T, Laatsch A, Rottke D, Rinninger F, Enrich C, Beisiegel U (2003). Recycling of apoprotein E is associated with cholesterol efflux and high density lipoprotein internalization. J Biol Chem.

[83] Rabut G, Ellenberg J, Goldman RD, Spector DL (2005). Photobleaching techniques to study mobility and molecular dynamics of proteins in live cells: FRAP, iFRAP and FLIP. Live Imaging: A Laboratory Manual.

