# Supplementary figures and images for: Annexin A6-Induced Alterations in Cholesterol Transport and Caveolin Export from the Golgi Complex
Source: Traffic. 2007 Nov;8(11):1568–89. doi: 10.1111/j.1600-0854.2007.00640.x (PMC3003291; doi:10.1111/j.1600-0854.2007.00640.x)

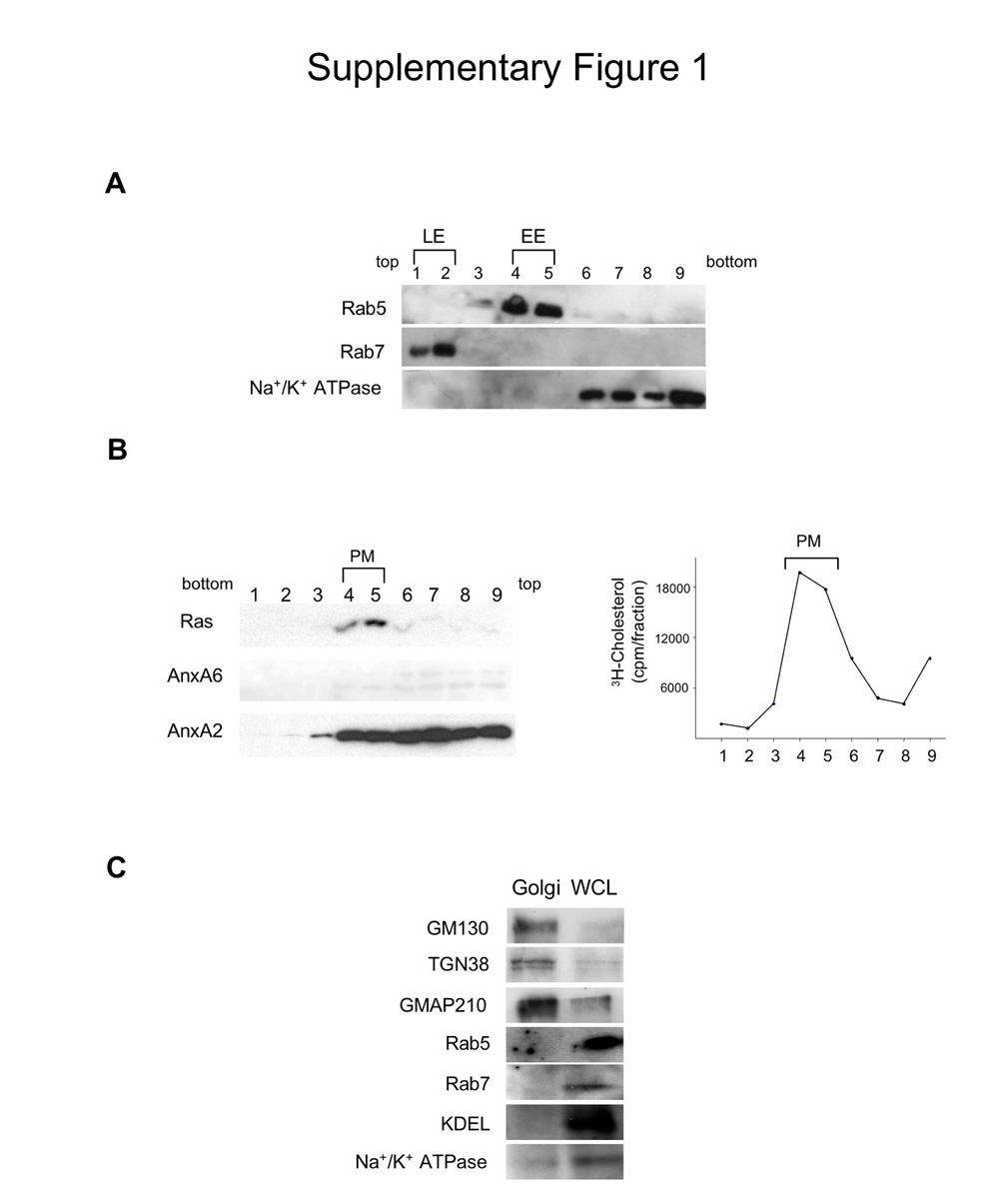

Supplement: Supplementary file 1 [file tra0008-1568-SD1.jpg]

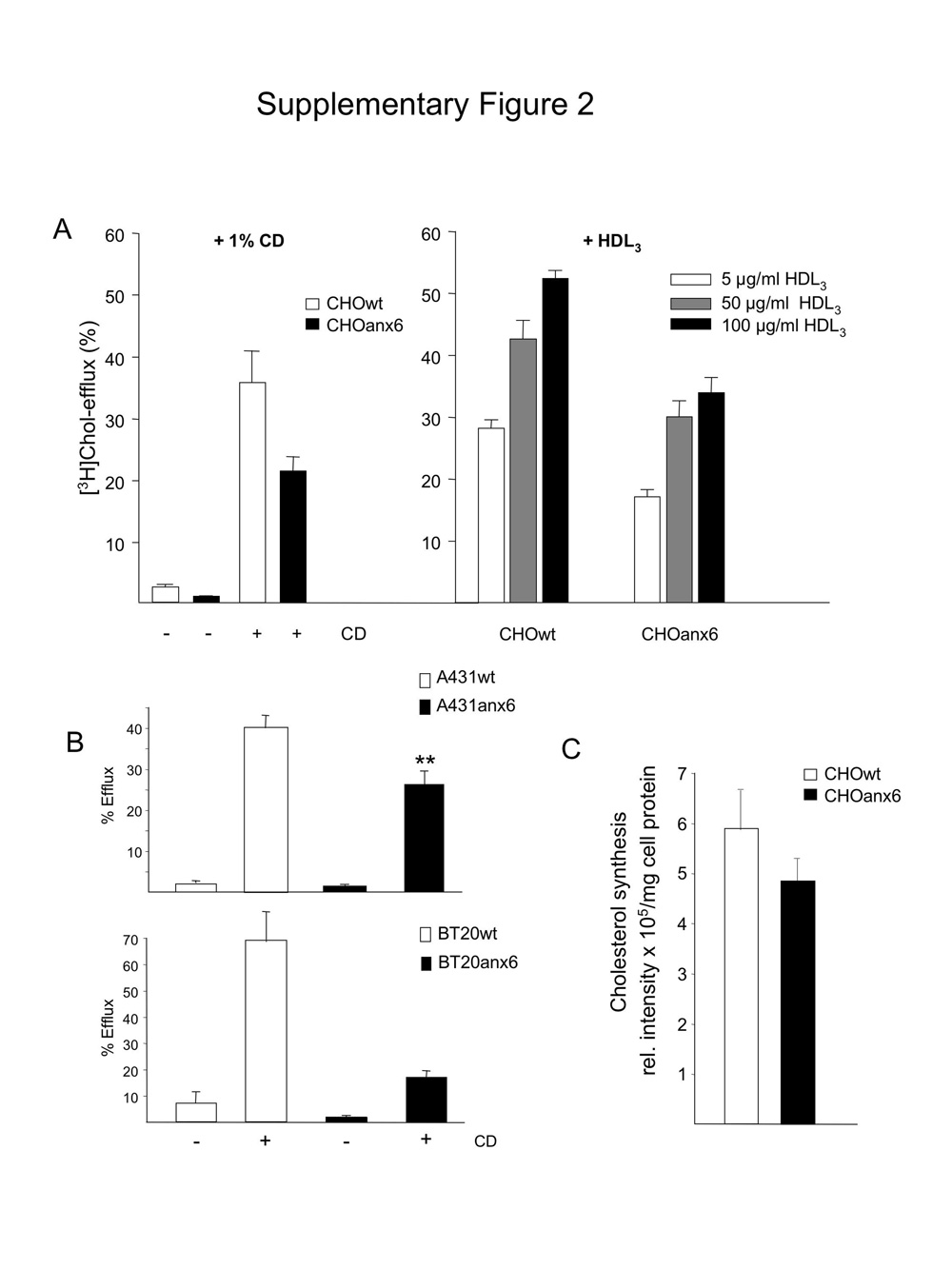

Supplement: Supplementary file 2 [file tra0008-1568-SD2.jpg]

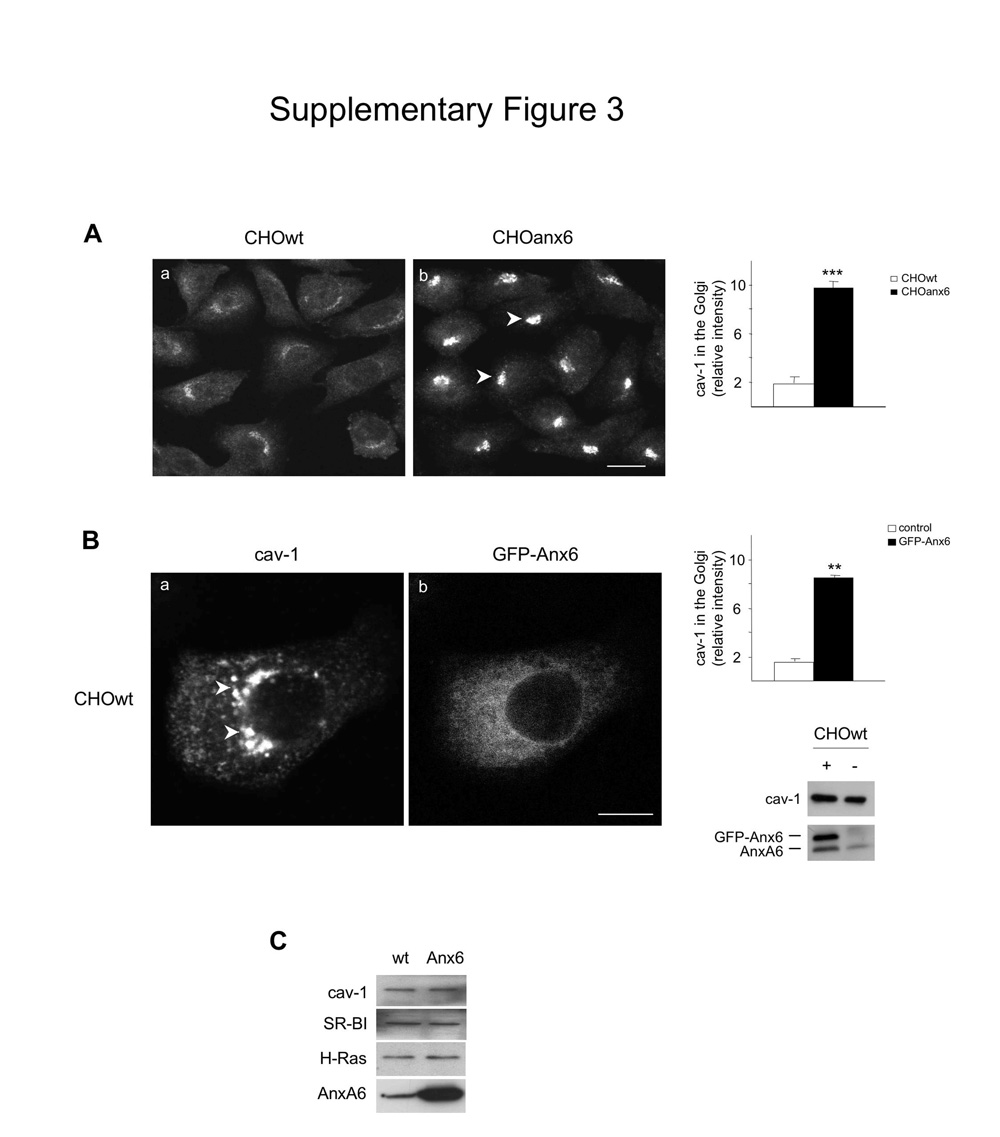

Supplement: Supplementary file 3 [file tra0008-1568-SD3.jpg]

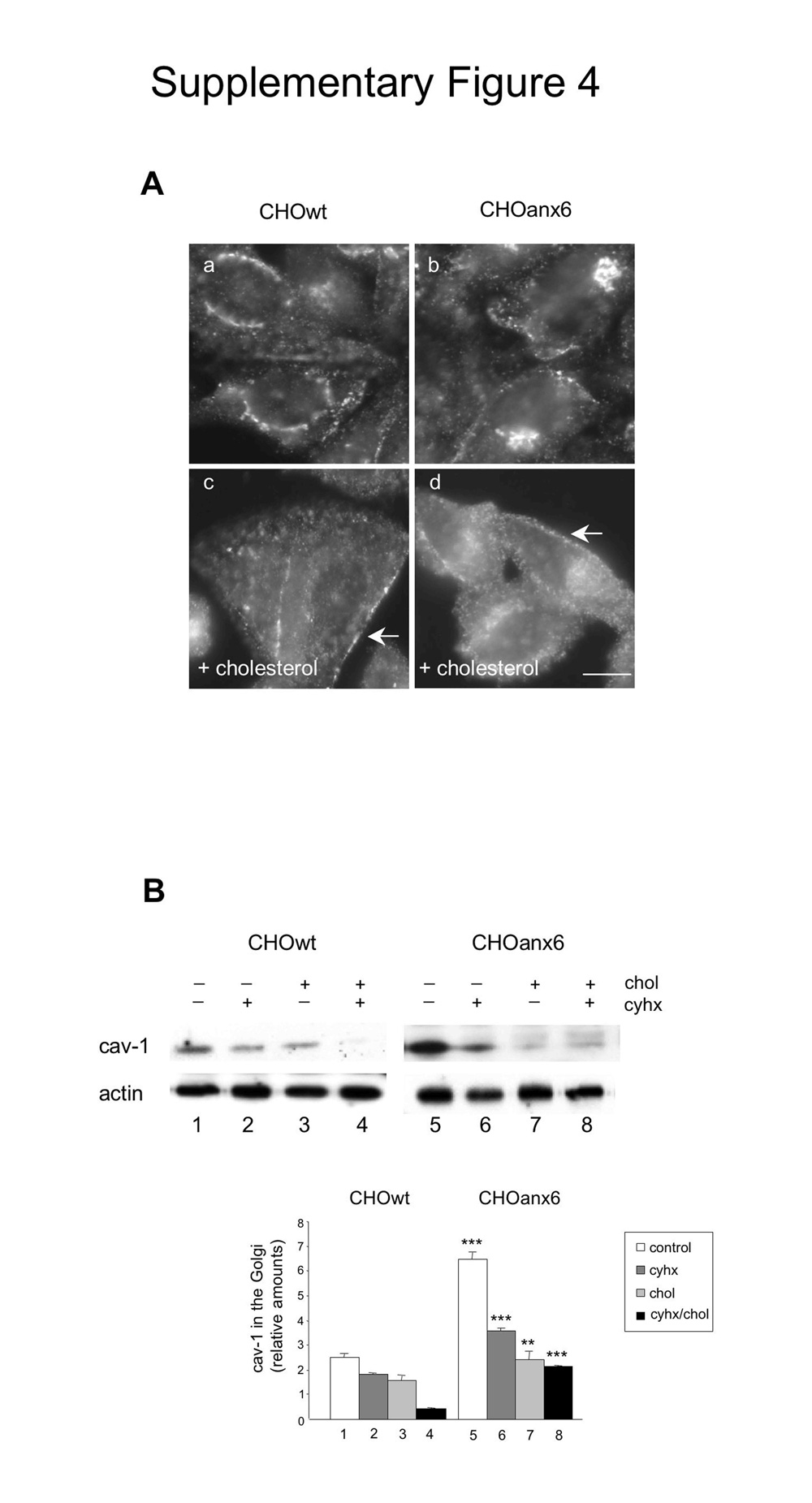

Supplement: Supplementary file 4 [file tra0008-1568-SD4.jpg]

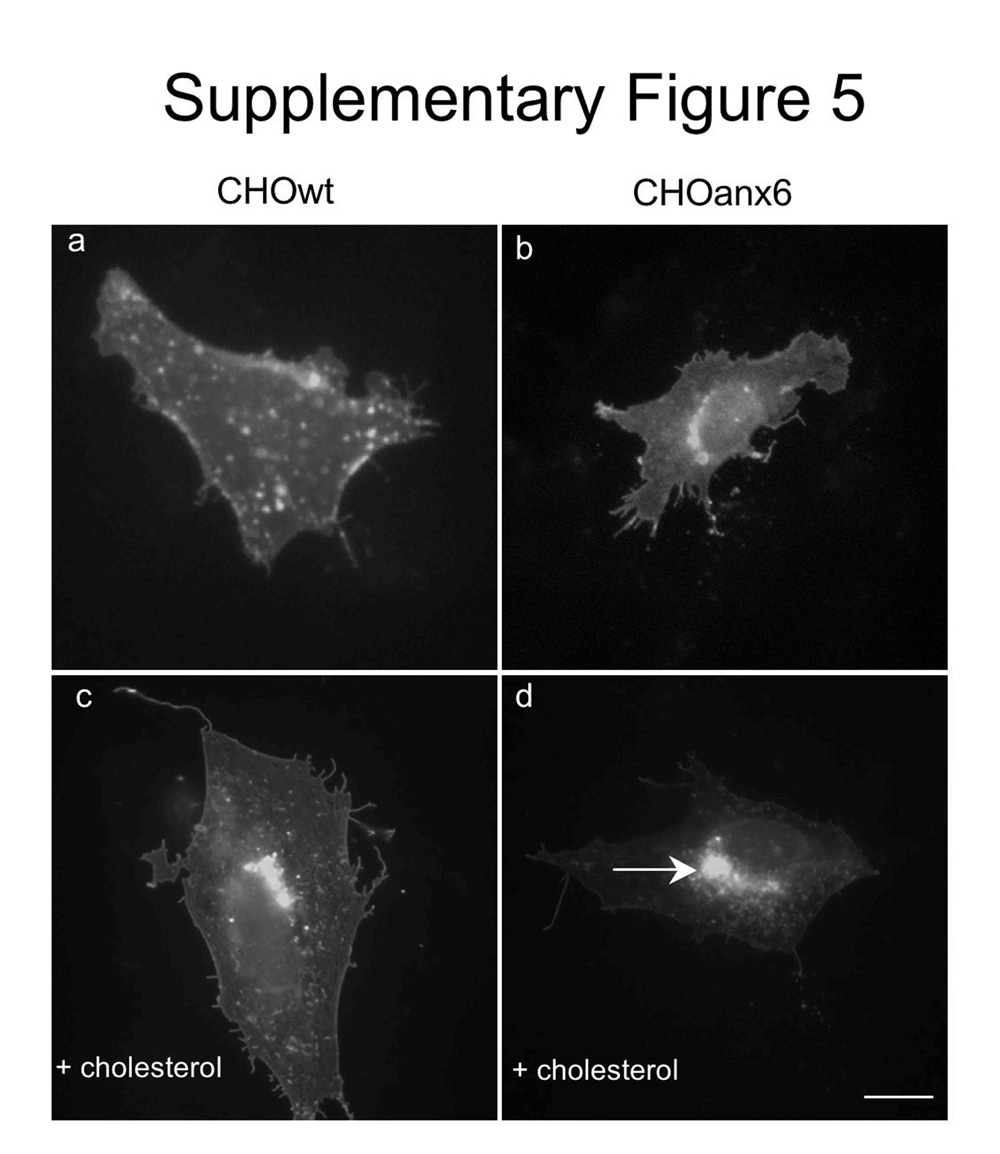

Supplement: Supplementary file 5 [file tra0008-1568-SD5.jpg]
